# The *Osiris* family genes function as novel regulators of the tube maturation process in the *Drosophila* trachea

**DOI:** 10.1371/journal.pgen.1010571

**Published:** 2023-01-23

**Authors:** Aaron Scholl, Istri Ndoja, Niraj Dhakal, Doria Morante, Abigail Ivan, Darren Newman, Thomas Mossington, Christian Clemans, Sruthi Surapaneni, Michael Powers, Lan Jiang

**Affiliations:** Department of Biological Sciences, Oakland University, Rochester, Michigan, United States of America; Northwestern University, UNITED STATES

## Abstract

*Drosophila* trachea is a premier model to study tube morphogenesis. After the formation of continuous tubes, tube maturation follows. Tracheal tube maturation starts with an apical secretion pulse that deposits extracellular matrix components to form a chitin-based apical luminal matrix (aECM). This aECM is then cleared and followed by the maturation of taenidial folds. Finally, air fills the tubes. Meanwhile, the cellular junctions are maintained to ensure tube integrity. Previous research has identified several key components (ER, Golgi, several endosomes) of protein trafficking pathways that regulate the secretion and clearance of aECM, and the maintenance of cellular junctions. The *Osiris* (*Osi*) gene family is located at the *Triplo-lethal* (*Tpl*) locus on chromosome 3R 83D4-E3 and exhibits dosage sensitivity. Here, we show that three *Osi* genes (*Osi9*, *Osi15*, *Osi19*), function redundantly to regulate adherens junction (AJ) maintenance, luminal clearance, taenidial fold formation, tube morphology, and air filling during tube maturation. The localization of Osi proteins in endosomes (Rab7-containing late endosomes, Rab11-containing recycling endosomes, Lamp-containing lysosomes) and the reduction of these endosomes in *Osi* mutants suggest the possible role of *Osi* genes in tube maturation through endosome-mediated trafficking. We analyzed tube maturation in zygotic *rab11* and *rab7* mutants, respectively, to determine whether endosome-mediated trafficking is required. Interestingly, similar tube maturation defects were observed in *rab11* but not in *rab7* mutants, suggesting the involvement of Rab11-mediated trafficking, but not Rab7-mediated trafficking, in this process. To investigate whether *Osi* genes regulate tube maturation primarily through the maintenance of Rab11-containing endosomes, we overexpressed *rab11* in *Osi* mutant trachea. Surprisingly, no obvious rescue was observed. Thus, increasing endosome numbers is not sufficient to rescue tube maturation defects in *Osi* mutants. These results suggest that *Osi* genes regulate other aspects of endosome-mediated trafficking, or regulate an unknown mechanism that converges or acts in parallel with Rab11-mediated trafficking during tube maturation.

## Author summary

The *Drosophila* trachea is the ideal model system to study conserved and fundamental mechanisms of tubular organ formation. In humans, tubular organs include, but are not limited to, the lungs, kidneys, and blood vessels. Defects in tube formation cause severe human diseases, such as polycystic kidney disease. After tubes begin to form, they will continue to mature to their functional lengths and diameters, and in the case of tracheae and lungs, will fill with air. The protein trafficking pathway, which is involved in delivering proteins to their final destinations, is heavily involved in various steps of the tube formation process. Previous studies have identified several key components within the protein trafficking pathway (ER, Golgi, endosomes) that regulate various processes during tube maturation. We identified the Osiris proteins, which are required for tube maturation including the maintenance of cellular junctions, clearance of the apical lumen, formation of taenidial folds to prevent the collapse of the tube, and air filling. In addition, Osi proteins regulate tube maturation potentially through endosome-mediated trafficking. This work will improve our understanding of the function of *Osi* genes and vesicular trafficking in the tube maturation process.

## Introduction

The *Drosophila* trachea is a premier model to study tube morphogenesis. It is a network of epithelial tubes with a monolayer of tightly-adhered polarized cells surrounding a central apical lumen. Previous research has revealed the mechanisms of the early steps of tube formation, including the specification of branch identities [1–9], the migration of tracheal cells [10–13], as well as branch fusion led by fusion cells at the tips of the branch to form an interconnected tubular network [14–18]. Tracheal tube maturation is a sequential process in which: (a) an apical secretion pulse deposits extracellular matrix components to form aECM [19]. This cable-like scaffold determines the shape of the tracheal tubes [20]; (b) the luminal aECM is degraded near the end of the embryogenesis [19], followed by the maturation of taenidial folds [21]. These chitin-containing helical strands run perpendicular to the tube length along the apical lumen to prevent the collapse of the tubes, especially after the clearance of the aECMs [21,22]. (c) The clearance of aECM and the establishment of osmotic pressure are the prerequisites of the following gas filling of the tracheal tubes [23,24]. All of these changes occur at the apical side of the tracheal tubes with minimal changes to the basolateral side [25].

Defective secretion of aECM often leads to tubes with inconsistent diameters and/or longer lengths [26–30]. Previous studies have identified components within the vesicular trafficking pathway involved in this process. Both COPII-mediated anterograde trafficking (ER to Golgi) and COPI-mediated retrograde trafficking (Golgi-to-ER) are required for the apical secretion of aECM [19,31–33]. In general, post-Golgi proteins are trafficked to their final destinations through endosomes [34]. Not surprisingly, several Rabs, which are often involved in endosome-mediated trafficking, have been implicated in tube maturation in the *Drosophila* trachea. For example, Rab9 and retromer are involved in the retrograde trafficking of the luminal protein, Serpentine (Serp) [35]; Rab35 mediates lumen formation in seamless tubes [36]; Rab39 is involved in apical lumen formation between fusion cells to form interconnected tubes [37];and Rab11 mediates the apical targeting of AJ component DE-cadherin (DE-cad) [38,39].

In addition to vesicular trafficking, intact cellular structures such as cell polarity and the cytoskeleton are also required for the secretion of luminal proteins. For instance, the loss of apical actin leads to the failed secretion of the luminal protein, Gasp [40]. Disrupting basolateral septate junctions (SJs), which are equivalent to vertebrate tight junctions, can also cause defective secretion of luminal proteins, such as Vermiform (Verm) and Serp [27,41–44].

The clearance of aECM depends on the clathrin-mediated endocytosis pathway [23,45]. Generally, apical proteins are internalized by clathrin-coated vesicles, which then fuse with the Rab5-containing early endosomes. The internalized proteins either travel through Rab7-containing multivesicular bodies (MVB)/late endosomes and eventually to lysosomes for degradation or they are recycled back apically or basally [46]. Disrupting the recruitment of clathrin to the apical membrane in *wurst* mutants abolishes lumen clearance and leads to longer tubes [23,45]. Taken together, previous research has identified several key components of the vesicular trafficking pathway (ER, Golgi, and several endosomes), which are required for *Drosophila* tracheal tube maturation. Here, we have identified the novel regulators of tracheal tube maturation, the *Osiris* (*Osi*) family genes.

The *Osi* gene family is primarily located within the *Triplo-lethal* (*Tpl)* locus on chromosome 3R 83D4-E3 and exhibits dosage sensitivity, including both haplo- and triplo-lethality [47]. These genes are predicted to have functional redundancy due to similar protein structures [48]. However, functional analyses of *Osi* genes are quite limited. Two studies indicate the potential role of *Osi* genes in endosome-mediated protein trafficking. In one case, RNAi knockdown of *Osi21* indicates its involvement in the lysosome-mediated degradation of endocytosed rhodopsin in the *Drosophila* eye [49]. Additionally, another study demonstrates that *Osi23* is essential for the formation of nanopores lining the olfactory sensillum in *Drosophila*, potentially through endo/lysosome-mediated trafficking [50]. However, the role of *Osi* genes in the *Drosophila* trachea is still unknown.

Previously, we identified seven *Osi* genes (*Osi9*, *Osi15*, *Osi17*, *Osi18*, *Osi19*, *Osi20*, *Osi24*) with obvious expression in the *Drosophila* trachea, beginning at stage 14 and continuing until the end of embryogenesis. Among these, five *Osi* genes (*Osi9*, *Osi15*, *Osi18*, *Osi19*, *Osi20)* have high levels of expression in the trachea. Expression of these HA-tagged *Osi* genes in the trachea using *btl-gal4* reveals that three of the five Osi proteins (HA-Osi9, HA-Osi15, HA-Osi19) are localized in vesicle-like structures in the cytoplasm and close to the apical membrane, whereas the remaining two Osi proteins (HA-Osi18, HA-Osi20) are highly concentrated at the apical membrane [51]. The vesicular localization of HA-tagged Osi9, Osi15, Osi19 suggests their potential functions in intracellular protein trafficking. The variations in protein localization also suggest a possible difference in functions among various *Osi* genes.

Here, we report that three *Osi* genes (*Osi9*, *Osi15*, *Osi19*) function redundantly to regulate tracheal tube maturation. No obvious defects are observed in single mutants, whereas apparent defects are observed in the double and triple mutant trachea, including discontinuous adherens junctions (AJs), retention of luminal components, disorganized taenidial folds, crushed tubes, and gas inflation defects. We also found that Osi proteins are mainly localized in endosomes (Rab7-containing late endosome, Lamp-GFP-containing lysosomes, Rab11-containing recycling endosomes). In addition, the numbers of these endosomes are reduced in the *Osi* mutant trachea. These observations suggest the probable involvement of *Osi* genes in endosome-mediated protein trafficking. Rab GTPases are factors that regulate endosome-mediated protein trafficking. Rab7 regulates the maturation of early endosomes to late endosomes, transport from early endosomes to late endosomes, and from late endosomes to lysosomes [52]. Rab11 is involved in the trafficking of internalized proteins back to the plasma membrane [53] as well as from the trans-Golgi network to the plasma membrane [54,55]. To test whether these endosomes contribute to tube maturation, we analyzed zygotic *rab11* and *rab7* loss of function mutants. Rab11 has been shown to mediate the apical targeting of AJ component DE-cad in *the Drosophila* trachea [38,39]. As expected, discontinuous AJs in *rab11* mutant trachea were observed. In addition, partial air filling and incomplete taenidial fold formation were also detected. Thus, *rab11*-mediated trafficking contributes to tube maturation. However, no obvious defects were observed in *the rab7* mutant trachea as well as in the trachea with overexpressed *rab7DN*. Thus, Rab7 may not play a significant role in tube maturation, however, we cannot rule out the minor roles that they might play.

To investigate whether *Osi* genes regulate tube maturation mainly through the maintenance of Rab11-containing recycling endosomes, we overexpressed *rab11* in *Osi* triple mutant trachea. Unexpectedly, no obvious rescue was observed. These results suggest that increasing the number of recycling endosomes is not sufficient for rescuing tube maturation defects in *Osi* mutants. *Osi* genes could be involved in other aspects of recycling endosome-mediated trafficking, such as potentially regulating the sorting and/or transporting of cargoes that contribute to tube maturation. Nevertheless, we cannot rule out the possibility that *Osi* genes regulate unknown pathways that converge or act in parallel with Rab11-mediated trafficking. In summary, this work reveals the novel functions of *Osi* genes in tube maturation.

## Results

### Osi proteins (Osi9, 15, 19) are expressed in vesicle-like structures in the *Drosophila* trachea

**Fig 1:**
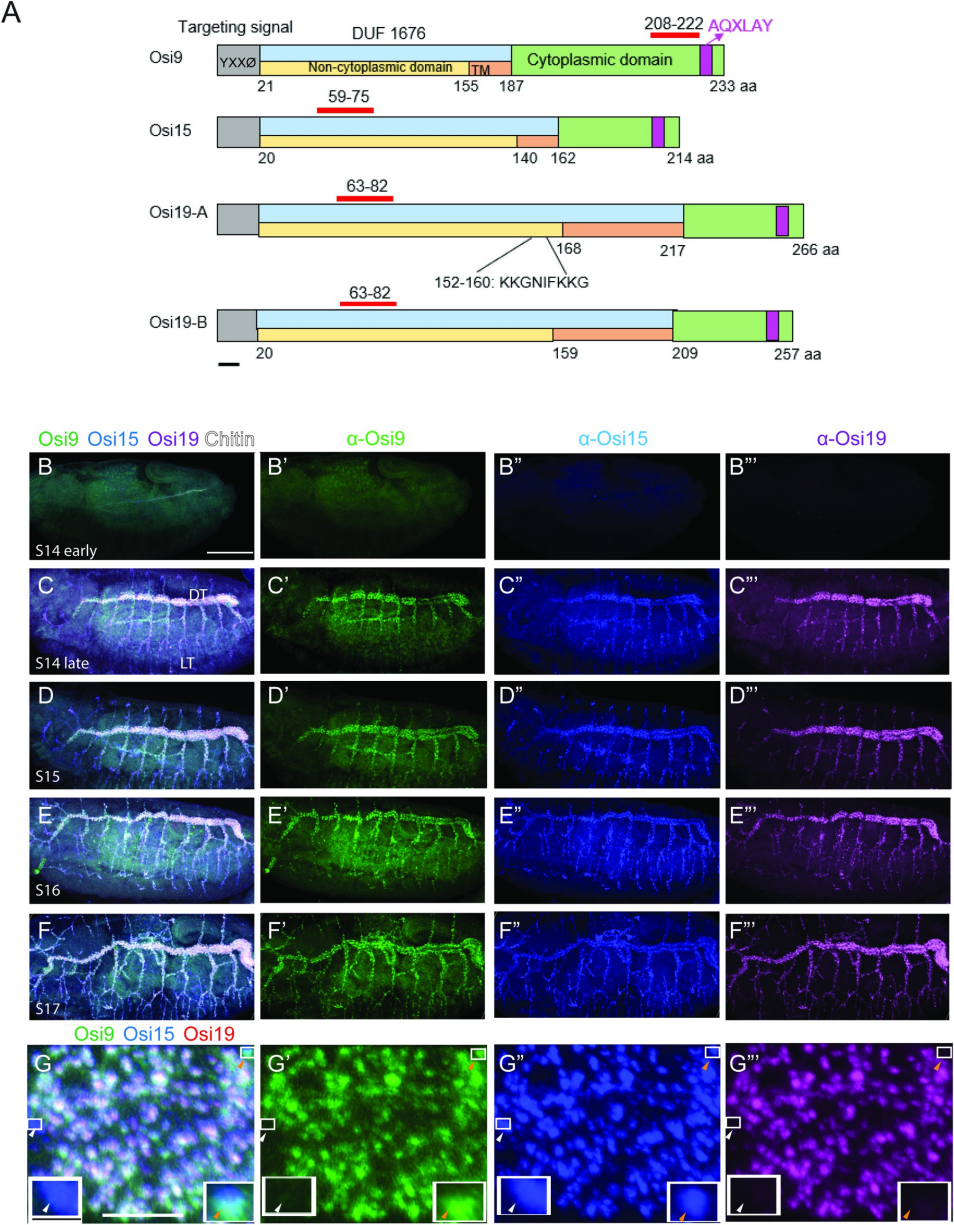
Osi proteins are expressed in vesicle-like structures in *the Drosophila* trachea. (A) Protein sequence annotation predicted multiple domains in Osi proteins. Predicted domains were: targeting signal containing YXXØ motif (grey), DUF 1676 domain (blue), non-cytoplasmic domain (yellow), transmembrane domain (orange), cytoplasmic domain (green), and an AQXLAY motif (purple). The red bars indicated the peptide sequence used to generate respective antibodies. The corresponding amino acids of those domains were indicated. Osi19 had two isoforms, Osi19-A and Osi19-B. The two isoforms were almost identical except that Osi19-A had extra 9 amino acids (KKGNIFKKG) which were missing from Osi19-B isoforms. The black bar in A represents 10 amino acids (aa). The localization of Osi9, Osi15, and Osi19 proteins was analyzed by immunostaining with Guinea pig anti-Osi9, Rat anti-Osi15, and Rabbit anti-Osi19 antisera as well as a chitin probe, which labels tracheal lumen using whole mount wild-type embryos. Osi9 (green), Osi15 (blue), and Osi19 (purple) proteins were expressed throughout the entire trachea from late-stage 14 till the end of embryogenesis but not at early-stage 14 when chitin was observed only in the dorsal trunk. Early-stage 14 (B-B‴), later stage 14 (C-C”’), stage 15 (D-D”’), stage 16 (E-E”’), and stage17 (F-F”’) embryos were shown. (G) Colocalization of Osi9 (green in G’), Osi15 (blue in G”), and Osi19 (purple in G”’) was shown where white and light purple depicted the colocalization of Osi9, Osi15, and Osi19 proteins. Two boxed areas in G-G‴ were enlarged in the left and right boxes in G-G‴. The white arrowheads showed an area that had Osi 15 expressed but not Osi9 and Osi19 expressions. The orange arrowheads showed an area with both Osi9 and Osi15 expressed, but without Osi19. The luminal matrix was depicted in white in the merged column (B-F). (White bar in B represented 50um and served as a reference for B-F”’. The white bar in G represented 10um and served as a reference for G-G”’. The black bar in the enlarged area in G represented 2um and served as a reference for the enlarged areas in G-G‴.

Osi family proteins show similar protein structures. The sequence annotation of Osi proteins revealed a targeting signal containing YXXØ motif, a transmembrane domain, a domain of unknown function DUF1676, and an AQXLAY motif (Fig 1A). YXXØ motif, where Y is tyrosine, X is any amino acid, and Ø is an amino acid with a bulky hydrophobic group, is the tyrosine-based motif that acts as a targeting signal for endosomal/lysosomal system as well as the basolateral membrane [56,57]. The specific function of the conserved AQXLAY motif has not been determined, though the Y in AQXLAY is the typical starting amino acid of the YXXØ motif. Nevertheless, the presence of these motifs in Osi proteins suggests their potential functions in vesicular trafficking.

Here, we focused on three *Osi* genes (*Osi9*, *Osi15*, and *Osi19*). The HA-tagged versions of these Osi proteins show similar vesicular localization in tracheal cells, suggesting potential functional redundancy [51]. To further confirm the intracellular localization of the endogenous Osi proteins, three Osi antibodies were generated in guinea pig, mouse, and rabbit respectively against specific regions in Osi9, Osi15, and Osi19 proteins (red lines in Fig 1A). These regions share no homology with other Osi proteins. Immunohistochemistry studies showed that Osi9 proteins (green in Fig 1C’–1F’) were distributed as numerous punctate structures throughout the entire trachea from late-stage 14 until the end of embryogenesis at stage 17. As expected, similar expression patterns of Osi15 (blue in Fig 1C”–1F”) and Osi19 (purple in Fig 1C’”–1F’”) were also observed. However, at early-stage 14, no obvious expression of all three Osi proteins was observed (Fig 1B–1B’”). Not surprisingly, almost complete co-localization of Osi9, Osi15, and Osi19 in vesicle-like structures was observed in segments of the dorsal trunk in stage 16 embryos (Fig 1G–1G”’), suggesting their potential redundant roles in vesicular trafficking. Notably, there were regions of immunostaining without all three Osi proteins present. For example, some punctae contained Osi15 but not Osi9 and Osi19 (Fig 1G–1G”’ left boxed area) whereas others contained Osi9 and Osi15 but not Osi19 (Fig 1G–1G”’ right boxed area). This subtle difference further confirms the specificity of the antibodies, as well as the potential minor differences in the functions between these proteins.

### Osi proteins (Osi9, 15, 19) are localized at endosomes

**Fig 2:**
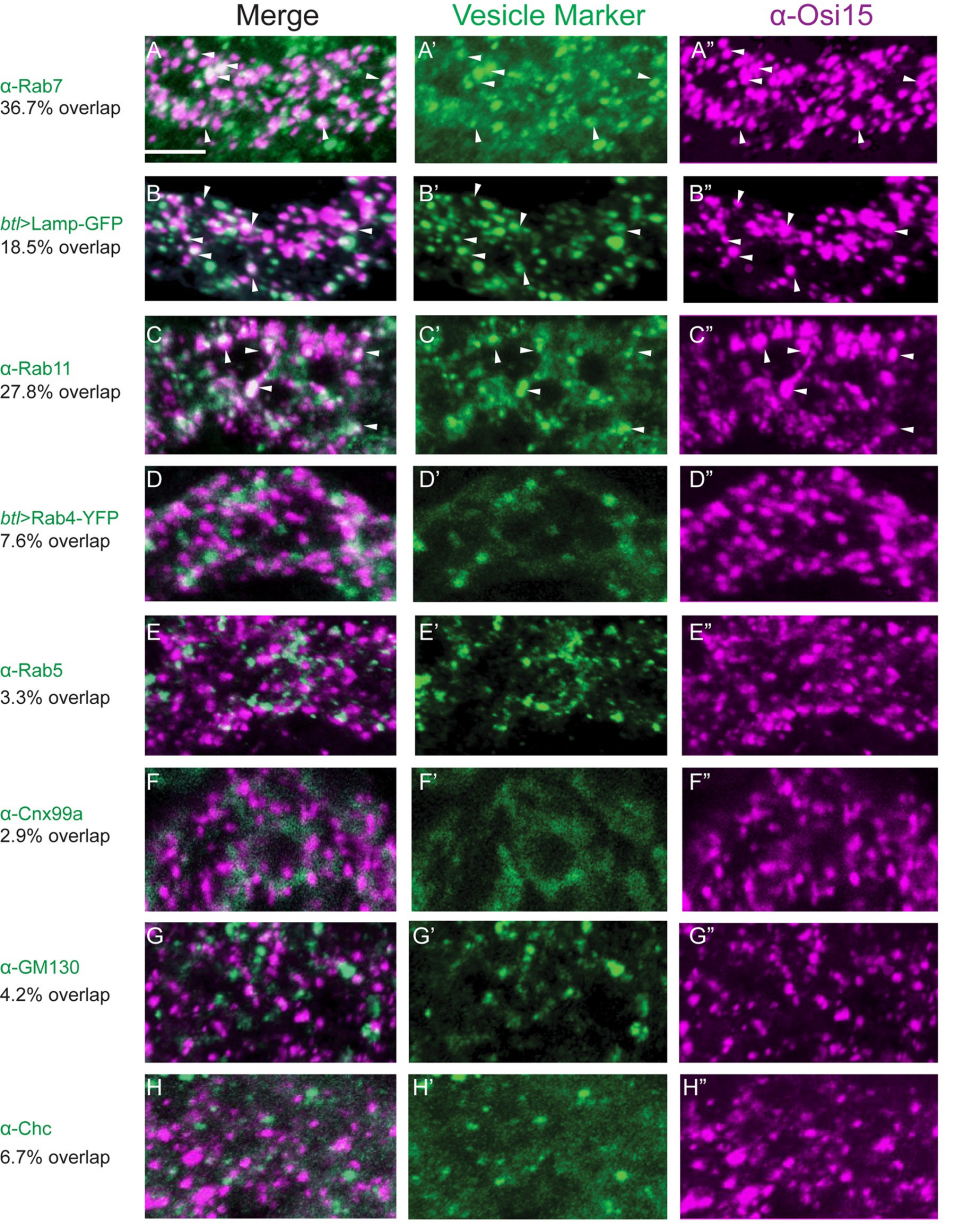
Osi proteins are localized in endosomes. Stage 16 embryos were co-immunostained with anti-Osi15 antisera (purple) and antibodies for respective markers (green) for ER, Golgi, and endosomes (Rab4, Rab5, Rab7, Rab11, and Lamp). Osi15 vesicles (purple in A”-C”) were partially colocalized with late endosome marker α-Rab7 (white arrowheads in A-A”), with lysosome marker btl>Lamp-GFP (white arrowheads in B-B”), with recycling endosome marker α-Rab11 (white arrowheads in C-C”). No significant co-localization between Osi15 vesicles (purple in D”-H”) and fast recycling endosome marker btl>Rab4-YFP (D-D”), early endosome marker α-Rab5 (E-E”), ER marker α-Cnx99a (F-F”), Golgi marker α-GM130 (G-G”), clathrin-coated vesicles marker α-Chc (H-H") was observed. Dorsal trunk (DT) segments were shown. The white line in A represented 5um and served as a reference for A-H". The percentage of Osi15 that colocalized with vesicle markers was shown.

The vesicular localization of Osi proteins suggests that Osi proteins are likely associated with the intracellular trafficking compartments. Therefore, we tested the colocalization of the Osi proteins with markers of components of the protein trafficking pathway. We analyzed the co-localization of Osi proteins (Osi9, Osi15, Osi19) with markers for ER (α-Cnx99a), Golgi (α-GM130), clathrin-coated vesicles (α-Chc), early endosome (α-Rab5), late endosome (α-Rab7), slow recycling endosome (α-Rab11), fast recycling endosome (*btl-gal4*, UAS-Rab4-YFP [58]), and lysosome (*btl-gal4*, UAS-Lamp-GFP [59]). Punctate Osi proteins (Osi15 was shown as an example) partially overlapped with the late endosome marker Rab7 (arrowheads in Fig 2A–2A”, 36.7% overlap; Pearson R = 0.67), lysosomal marker Lamp-GFP (arrowheads in Fig 2B–2B”, 18.5% overlap; Pearson R = 0.45), and with recycling endosome marker Rab11 (arrowheads in Fig 2C–2C”, 27.8% overlap; Pearson R = 0.61). However, there was only sporadic co-localization with fast recycling endosome *btl-gal4*, UAS-Rab4-YFP (Fig 2D–2D”, 7.6% overlap; Pearson R = 0.48), with early endosome marker Rab5 (Fig 2E–2E”, 3.3% overlap; Pearson R = 0.16), with ER marker Cnx99a (Fig 2F–2F”, 2.9% overlap; Pearson R = 0.29), with Golgi marker GM130 (Fig 2G–2G”, 4.2% overlap; Pearson R = 0.37), and with clathrin-coated vesicle marker Chc (Fig 2H–2H”, 6.7% overlap; Pearson R = 0.37).

Rab7 regulates the maturation of early endosomes to late endosomes, transport from early endosomes to late endosomes, and from late endosomes to lysosomes [52]. Rab11 is involved in the trafficking of internalized proteins back to the plasma membrane [53] as well as from the trans-Golgi network to the plasma membrane [54,55]. The co-localization of Osi proteins with these endosomes suggests the involvement of Osi proteins in endosome-mediated protein trafficking within the *Drosophila* trachea.

### Generation of *Osi* loss of function mutants by CRISPR

**Fig 3:**
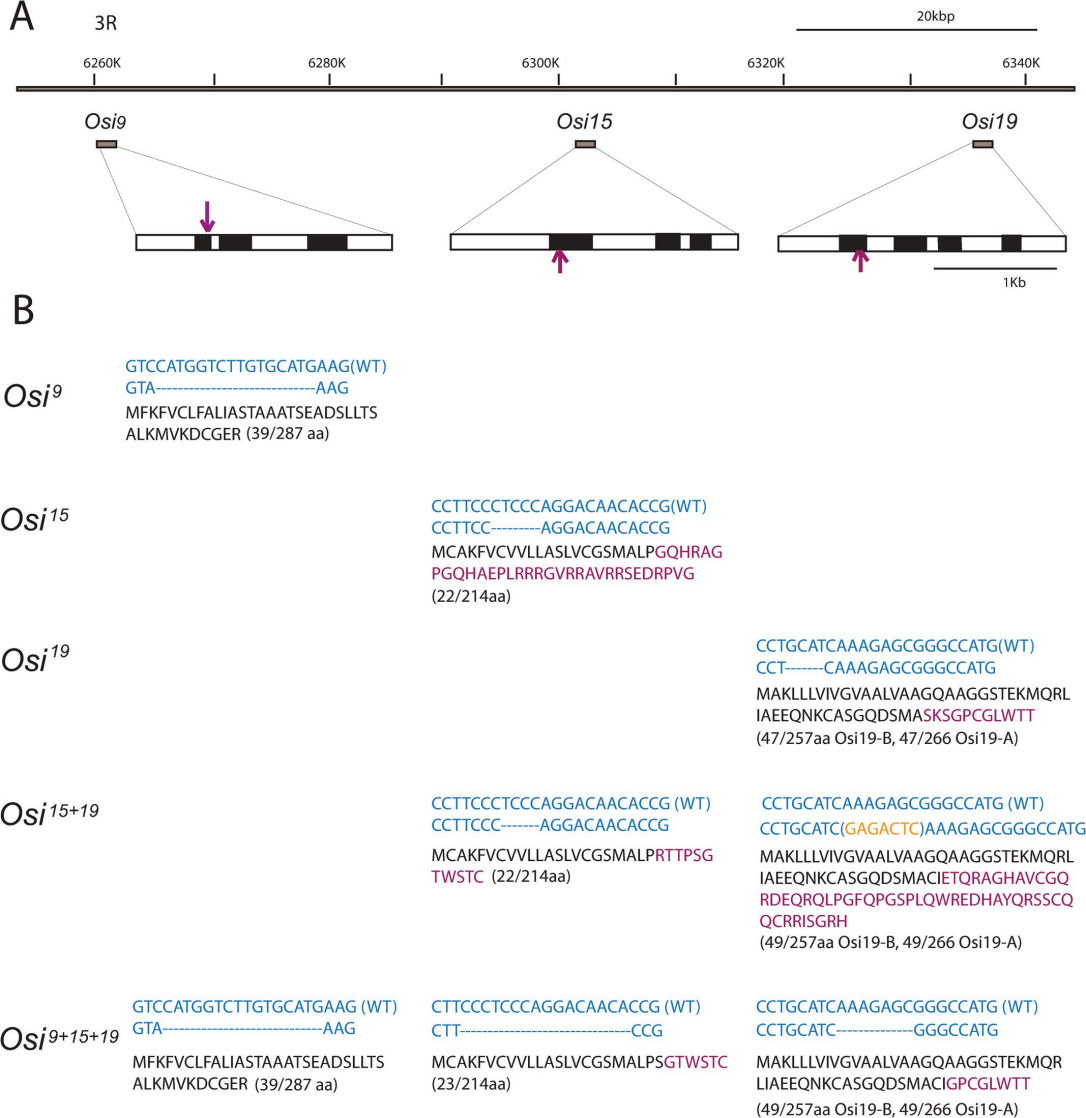
Generation of *Osi* loss of function mutants. Osi loss of function mutants were generated by CRISPR-Cas9. Single mutants *Osi*^*9*^, *Osi*^*15*^, and *Osi*^*19*^, double mutants *Osi*^*15+19*^, and triple mutants *Osi*^*9+15+19*^ were shown. The genomic locus for *Osi9*, Osi15, and *Osi19* were displayed in (A) including intron (white box) and exon regions (black box). The CRISPR target site selected for each was indicated by a purple arrow. (B) *Osi*^*9*^, *Osi*^*15*^, *Osi*^*19*^, *Osi*^*15+19*^, and *Osi*^*9+15+19*^ mutants were described with blue text representing the genomic sequence and black text representing the translated protein sequence. The partial sequence near the target site for each wild-type (WT) *Osi* gene was listed as the top line of blue text, while the mutant sequence was listed as the bottom line of blue text. In the CRISPR-modified genomic sequences, "-" represented deleted bases and yellow text in parentheses represented inserted bases. The translated protein sequences of the mutants were listed in black and purple text beneath the genomic sequences. The black text represented amino acids that match wild-type protein sequences, while purple amino acids were the result of frameshifts introduced during CRISPR genome editing. Following each amino acid sequence, the intact amino acids in the mutant sequences were compared to the original number of amino acids in the respective Osi proteins.

While evidence points to the likelihood of the involvement of *Osi* genes in vesicular trafficking, the functions of these genes in the trachea have not been studied. Therefore, we generated *Osi* loss of function mutants. The potential functional redundancy of *Osi* genes prompted us to generate *Osi* single, double, and triple loss-of-function mutants using CRISPR-Cas9. All three *Osi* genes are located in chromosome 3R (Fig 3A). Using a single gRNA line that targets both *Osi15* and *Osi19* genes, we generated an *Osi15* single mutant (*Osi*^*15*^), encoding 22 of the original 214 aa (22/214) of Osi15 protein; an *Osi19* single mutant (*Osi*^*19*^), encoding 47/266 of the Osi19-A and 47/257 of Osi19-B proteins, respectively; an *Osi15* + *Osi19* double mutant (*Osi*^*15+19*^) encoding 22/214 of *Osi15*, and 49/266 aa of Osi19-A and 49/257 of Osi19-B proteins, respectively (Fig 3B). In addition to the regions near the target sites, the regions between the *Osi15* and *Osi19* genes were also amplified to ensure that these regions were intact. Using a similar approach, we generated a severely truncated *Osi9* single mutant (*Osi*^*9*^), encoding 39/287aa of *Osi9* protein (Fig 3B). The *Osi9* + *Osi15* + *Osi19* triple mutant (*Osi*^*9+15+19*^), encoding 39/287 of Osi9 protein, 23/214 of the Osi15 protein, and 49/266 of Osi19-A and 49/257 of Osi19-B proteins, respectively, was isolated using the same gRNA carrying the targeting sequences of *Osi15* and *Osi19* in *Osi*^*9*^ mutant background (Fig 3B). Again, the regions between *Osi15* and *Osi19* in the *Osi* triple mutant were checked via PCR and Sanger sequencing to ensure that these regions remained intact. Therefore, these mutants only carried deletions in the respective genes and left other genes intact. The expression of these Osi proteins was then analyzed in these mutants by immunohistochemistry.

Unlike in wild-type control, where all three Osi proteins were expressed in the trachea (S1A–S1A”’ Fig), Osi9 protein was not observed whereas Osi15 and Osi19 proteins were present in single mutant *Osi*^*9*^ (S1B–S1B”’ Fig). Similarly, both Osi9 and Osi19 except Osi15 proteins were present in single mutant *Osi*^*15*^ (S1C–S1C”’ Fig). Likewise, both Osi9 and Osi15 but Osi19 proteins were present in single mutant *Osi*^*19*^ (S1D–S1D”’ Fig). Also, neither Osi15 nor Osi19 proteins were present in *Osi*^*15+19*^ double mutant whereas Osi9 was expressed (S1E–S1E”’ Fig). As expected, in *Osi*^*9+15+19*^ triple mutants none of the three proteins were observed (S1F–S1F”’ Fig). These results confirmed that the *Osi* mutants are null or severe loss of function mutants and also indicated the specificity of the antibodies.

### *Osi* genes (*Osi 9*, *15*, *19*) are required for gas filling and tube morphology in *the Drosophila* trachea

**Fig 4:**
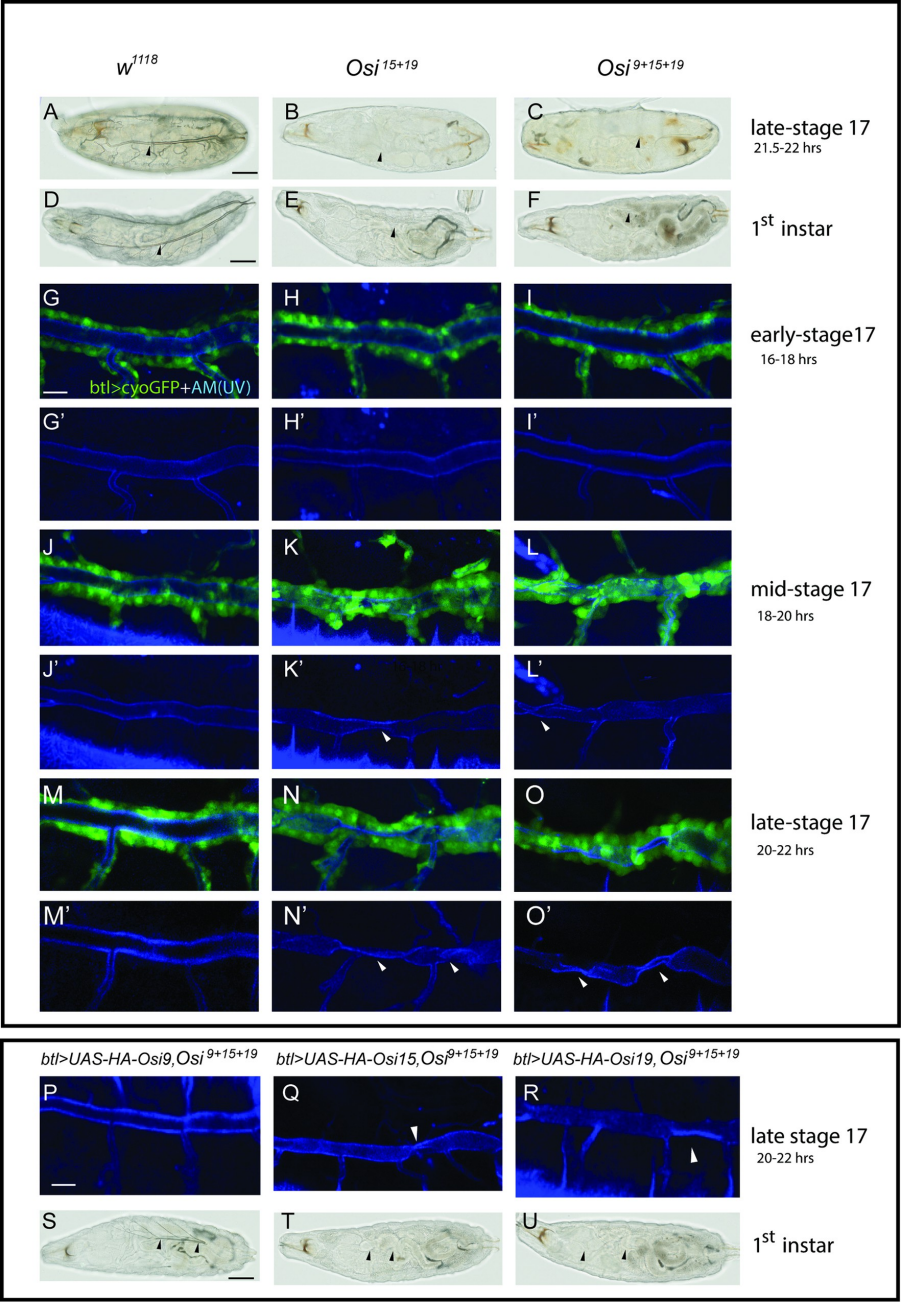
*Osi* genes are required for tube morphology and tracheal gas filling. (A, D) Brightfield microscopy images showed the wild-type tracheae were filled with gas (arrowheads) in late-stage 17 (30min before hatching) and 1^st^ instar larvae. In *Osi*^*15+19*^ double (B, E) and *Osi*^*9+15+19*^ triple (C, F) mutants, tracheal tubes were not visibly filled with air (arrowheads). (G-O’) Tracheal cells in early (16–18 hrs after egg laying), mid (18-20hrs after egg laying), and late-stage 17 (20–22 hrs after egg laying) embryos were outlined in green by the tracheal cell cytoplasmic marker btl>cyto GFP while the apical membrane (AM) was shown in blue by UV illumination. (G-G’, J-J’, M-M’) In early, mid, and late-stage 17, wild-type tracheae exhibited dorsal trunks of uniform size and shape without apparent defect. At early-stage 17, *Osi*^*15+19*^ double (H-H’) and *Osi*^*9+15+19*^ triple (I-I’) mutants showed similar dorsal trunk features when compared to the wild type. By mid-stage 17, *Osi*^*15+19*^ double (K-K’) and *Osi*^*9+15+19*^ triple (L-L’) mutants exhibited crushed tracheal apical membranes (arrowheads). By late-stage 17, *Osi*^*15+19*^ double (N-N’) and *Osi*^*9+15+19*^ triple (O-O’) mutants exhibited severe crushed tracheal apical membranes (arrowheads). (P, S) Overexpression of *Osi9* in the triple mutant background (*btl>UAS-HA-Osi9*, *Osi*^*9+15+19*^) significantly rescued the tube structure defect and partially rescued the air filling defect. Overexpression of either *Osi15* (*btl>UAS-HA-Osi15*, *Osi*^*9+15+19*^) (Q, T) or *Osi19* (*btl>UAS-HA-Osi19*, *Osi*^*9+15+19*^) (R, U) respectively in triple mutant backgrounds were each not sufficient for air filling rescue. Notably, *Osi15* (Q) and *Osi19* (R) overexpression in the triple mutant background each demonstrated a partial structural rescue of the apical membrane when compared to the triple mutant, *Osi*^*9+15+19*^ (O’). The black scale bar in A, D, and S represented 50 μm and served as a reference for A-F, S-U. The white scale bar in G and P represented 10 μm and served as a reference for G-O’, P-R.

We analyzed the survival rate of *Osi* mutants. Single mutants *Osi*^*9*^ *and Osi*^*15*^ were homozygous viable, whereas *Osi*^*19*^ homozygous mutants were semi-lethal (N = 60, all survived to the pupal stage, and 10% survived to adults). Unlike air-filled wild-type control (Fig 4A), both *Osi*^*15+19*^ (Fig 4B) and *Osi*^*9+15+19*^ (Fig 4C) mutant trachea completely lacked gas filling in late-stage 17 embryos, 30min before hatching (21.5–22 hrs after egg laying). These mutant embryos hatched as 1^st^ instar larvae about 22 hrs after egg laying, similar to the wild-type control. The mutant larvae continued to survive 2–3 days with minimal growth while the wild-type control larvae developed to the 2^nd^ and 3^rd^ instar stage within the same time frame. This air-filling defect was 100% penetrant in both the embryonic and larval stages (Compare Fig 4E and 4F to 4D).

To visualize tracheal morphology, tracheal cells were outlined by cytoplasmic-GFP, which was expressed in the trachea using *btl-gal4* [60]. Meanwhile, the apical membrane was visualized by UV light (blue). Since the tube sizes at the posterior tracheal segments are relatively larger and easier to observe, the tracheal segments 7–9 were analyzed. At early-stage 17 (16–18 hrs after egg laying), no obvious changes were observed in *Osi*^*15+19*^ and *Osi*^*9+15+19*^ mutant trachea compared to wild type (compare Fig 4H–4H’ and 4I–4I’ with 4G–4G’ respectively). At mid-stage 17 (18–20 hrs after egg laying), in contrast to wild-type tubes (Fig 4J and 4J’), crushed tubes started to appear in double mutant *Osi*^*15+19*^ (arrowhead in Fig 4K and 4K’) and triple mutant *Osi*^*9+15+19*^ trachea (arrowhead in Fig 4L and 4L’). At late-stage 17 (20–22 hrs after egg laying), the tubes were crushed more severely (compare Fig 4N–N’ and 4O–4O’ with 4M–4M’ respectively). To test whether these defects are due to the loss of *Osi* genes, we overexpressed individual HA-tagged *Osi* gene [51] in *Osi*^*9+15+19*^ mutant trachea by *btl-gal4* and analyzed the rescue effect. *Osi9* overexpression significantly rescued the crushed tube defects (Fig 4P) and partially rescued the gas-filling defects (Fig 4S). However, overexpression of either *Osi15* or *Osi19*, respectively, only partially rescued the tube morphology defect, as sections of crushed tubes were still observed (arrowheads in Fig 4Q and 4R), and failed to rescue the air inflation defect (Fig 4T and 4U). This variation is likely not due to the difference in the levels of expression, since these transgenes were expressed at similar levels (S2A–S2C Fig). Nevertheless, the significant or partial rescue by individual *Osi* genes suggests that *Osi* genes have functional redundancy in the trachea. However, we cannot rule out the possible minor functional difference between these genes. Since *Osi9* shows the most efficient rescue, we used this line to rescue other defects observed in *Osi*^*9+15+19*^ mutant trachea for the following experiments.

### *Osi* genes are required for the complete clearance of aECM and taenidial folds formation

The defective gas filling and crushed tube phenotypes in *Osi*^*9+15+19*^ mutants encouraged us to analyze the process of tube maturation. An efficient apical secretion pulse leads to the formation of aECM from stages 14 to 16. This first wave of aECM is cleared from the lumen by an endocytic wave during mid-stage 17 [61]. The clearance of luminal ECM is followed by the maturation of taenidial folds, a helical structure, which starts to develop at late embryonic stage 15 and matures at embryonic mid-stage 17 [21]. The taenidial folds prevent the collapse of the tracheal tubes [22]. Thereafter, the liquid in the lumen is cleared and followed by gas filling. During tube maturation, the initiation of each change is coupled with the completion of the previous steps in a strict developmental sequence. For instance, the defective early secretion of the luminal matrix in Sar1 mutants leads to luminal clearance defects [62]; the clearance of solid aECM material and the liquid within the tubes are prerequisites for the gas filling of the tracheal tubes [23,24]; and defects in taenidial folds stability also leads to crushed tubes and air filling defect [63]. Thus, we analyzed the early secretion and clearance of aECM, as well as the taenidial fold formation in *Osi* mutants.

**Fig 5:**
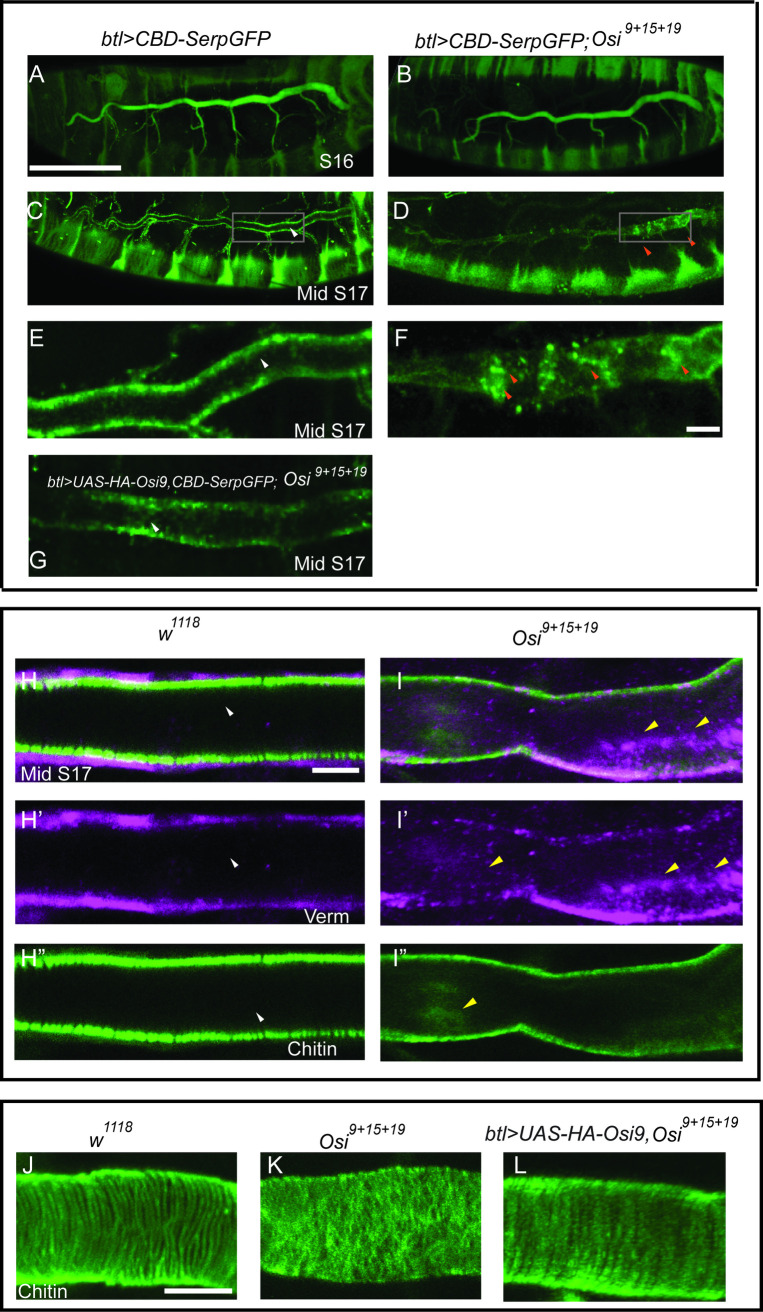
*Osi* genes are required for the complete clearance of aECM and the establishment of taenidial folds. The GFP-tagged chitin-binding domain (CBD) of Serp was expressed in the trachea using *btl-gal4*. Mid-stage17 (19.5–20 hrs after egg laying) embryos were used to analyze luminal clearance and taenidial fold formation. (A, B) The secretion of Serp at stage 16 in the *Osi*^*9+15+19*^ triple mutant was consistent with wild-type secretion. (C-D) By mid-stage 17 in *Osi*^*9+15+19*^ triple mutants, Serp (orange arrowheads) was not completely cleared from the lumen, which was most obvious at posterior segments when compared to cleared Serp (white arrowheads) in wild-type trachea of the same stage. (E, F) enlarged boxed area in C and D. (G) *Osi9* overexpression in the triple mutant background (*btl>UAS-HA-Osi9*, *Osi*^*9+15+19*^) at mid-stage 17, Serp was mostly cleared from the lumen, similar to wild type mid-stage 17 embryos (E). (H-I) Mid-stage 17 wild type and *Osi*^*9+15+19*^ triple mutant embryos were immunostained with Chitin (green) and Verm (purple) to illustrate luminal clearance defects. The yellow arrowheads in (I-I”) and white arrowheads in (H-H”) showed luminal components that remained in the lumen of *Osi*^*9+15+19*^ triple mutant trachea and that were cleared from the lumen in wild-type control respectively. (J) Wild-type taenidial folds at mid-stage 17 appeared organized and well-defined, as indicated by the chitin-binding probe (in green). (K) *Osi*^*9+15+19*^ triple mutants lacked defined tracheal taenidial folds when compared to the wild type. (L) Overexpression of *Osi9* in the triple mutant background (*btl>UAS-HA-Osi9*, *Osi*^*9+15+19*^) appeared to significantly rescue the taenidial fold defects seen in triple mutants. However, these taenidial folds were less defined when compared to the wild-type control. The white scale bar in A represented 100 μm and served as a reference for A-D. The white scale bars in F, H, and K represented 10 μm and served as references for E-G, H-I", K-M respectively.

The chitin-based luminal matrix contains chitin, chitin modifying enzymes, including Verm and Serp [27] as well as chitin-binding proteins, including Obstructor A (ObstA) [28] and Gasp [26]. Tracheal expression of the GFP-tagged chitin-binding domain of Serp (CBD-Serp-GFP) shows similar secretion and clearance as the endogenous luminal proteins in wild-type trachea [64]. Thus, we expressed CBD-Serp-GFP in *Osi*^*9+15+19*^ mutants using *btl-gal4* to analyze the secretion and clearance of luminal proteins. At stage 16, similar to the wild-type control (Fig 5A), secretion of CBD-Serp-GFP into the lumen in *Osi*^*9+15+19*^ mutant trachea (Fig 5B) was observed. A luminal clearance occurs at mid-stage 17 around 18 hrs 48min after egg laying [19]. Therefore, we analyzed mid-stage 17 embryos that are 19.5–20 hrs after egg laying. The luminal CBD-Serp-GFP was cleared from the lumen in wild-type trachea (white arrowheads in Fig 5C and enlarged area in Fig 5E). Although most of the luminal CBD-Serp-GFP was cleared in *Osi*^*9+15+19*^ mutant trachea, some residual proteins remained in the lumen, mostly at the posterior segments (orange arrowhead in Fig 5D and enlarged area in 5F). To further confirm that *Osi* genes are required for the clearance of luminal components, we analyzed luminal protein components Verm and Chitin in mid-stage 17 embryos (19.5–20 hrs after egg laying) by immunostaining. Residual Verm and Chitin were still visible in the lumen in *Osi*^*9+15+19*^ mutant trachea (yellow arrowheads in Fig 5I–5I”) whereas these components were cleared from the lumen in wild-type trachea (white arrowheads in Fig 5H–5H"). Thus, in *the Osi* mutant trachea, although a majority of the luminal components were cleared, some residual luminal components remained.

Taenidial folds start to develop at late embryonic stage 15 and mature at mid-stage 17 when aECM is cleared from the lumen [21]. These helical strands provide tube integrity as well as flexibility [22]. Unlike in wild-type trachea where the taenidial folds were arranged in parallel helical strands (Fig 5J), the taenidial folds in *Osi*^*9+15+19*^ mutant trachea appeared disorganized and undefined (Fig 5K). As expected, both the luminal clearance (Fig 5G) and taenidial folds (Fig 5L) defects in *Osi*^*9+15+19*^ mutant trachea were significantly rescued by *Osi9* overexpression. Thus, *Osi* genes are required for complete luminal clearance and taenidial fold formation.

### *Osi* genes are required for the stability of AJs

**Fig 6:**
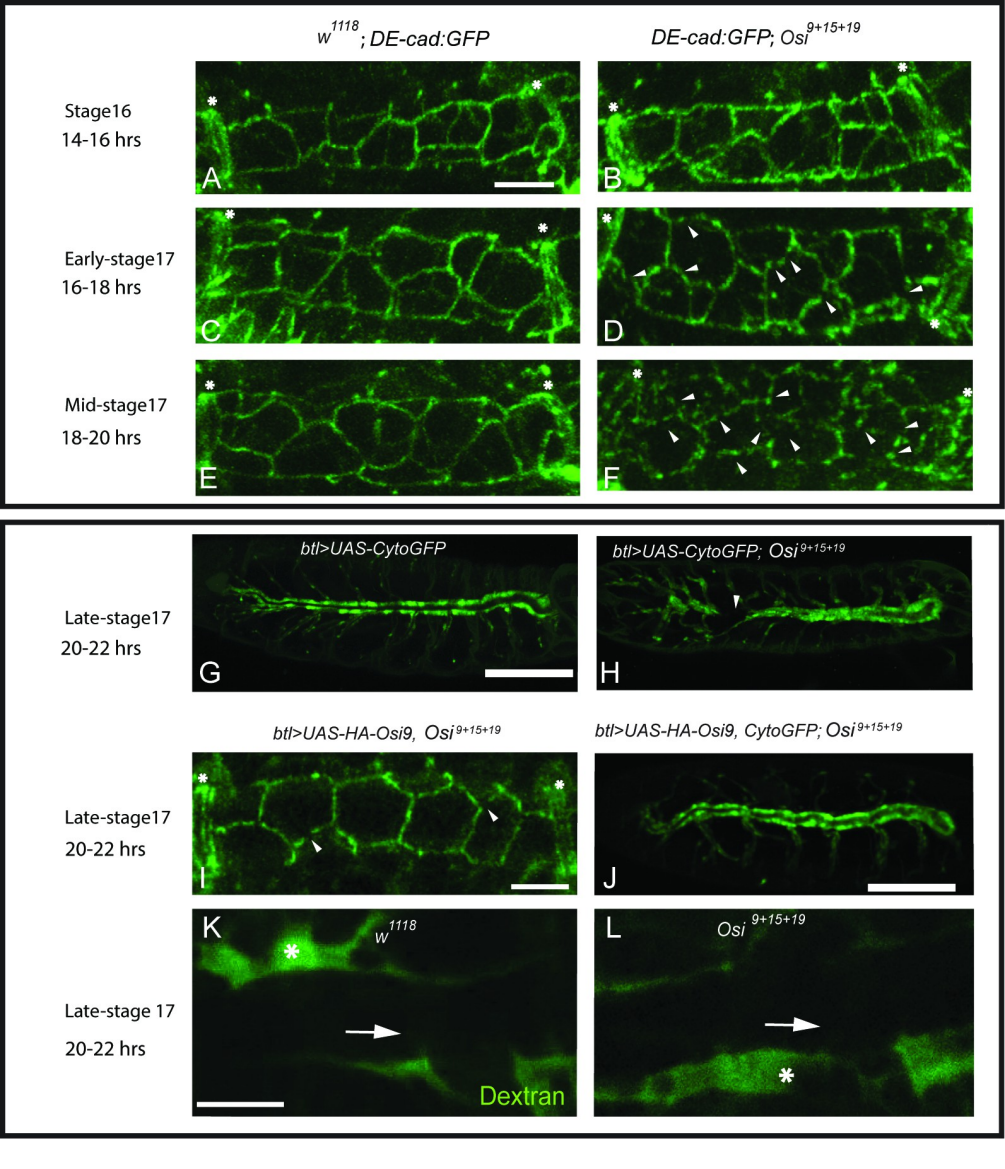
*Osi* genes are required for the maintenance of the AJs but not SJs. (A, C, E) Wild type DE-cad:GFP expression in green showed uniform localization along AJs throughout stage 16 and stage 17. (B, D, F). DE-cad:GFP expression in the *Osi*^*9+15+19*^ triple mutant started to become discontinuous (breaks) at early-stage 17, and more breaks in each AJ were observed at mid-stage 17. (D, F) White arrows in each image pointed to breaks in the continuity of DE-cad:GFP expression. (A-F, I) White asterisks indicated fusion cell locations. (G-H) The cytoplasm of tracheal cells was outlined by btl>Cyto GFP at late-stage 17 embryos. (G) In wild-type embryos, there was a distinct region where the lumen was located. (H) In *Osi*^*9+15+19*^ triple mutant, one or two tracheal gaps (white arrowhead) were observed on average. Attempts to rescue through *Osi9* overexpression in the triple mutant background (*btl>UAS-HA-Osi9*, *Osi*^*9+15+19*^) (I) showed DE-cad:GFP expression was rescued to near-wild type continuity with only a few breaks (white arrowheads) and (J) no obvious tracheal gaps were observed. (K-L) Dextran-FITC injection into late-stage 17 *Osi*^*9+15+19*^ triple mutant and wild-type embryos showed that there were no defects in the barrier function of the SJs. Asterisks showed the neighboring tissues with dye present, while the white arrows pointed to the trachea that was devoid of dextran-FITC. The white scale bar in (A, I, K) represented 10 μm and served as references for A-F, I, and K-L respectively. The white scale bar in (G, J) represented 100 μm and served as references for G-H, and J respectively.

The tube defects in *Osi* mutant trachea also prompted us to analyze the possible disruption of the cellular junctions in *the Osi*^*9+15+19*^ mutants. AJs mediate cell-cell adhesion between neighboring cells. AJs contain the cell-adhesion molecule DE-cad and its cytoplasmic binding partners, β-catenin and α-catenin. The maintenance and remodeling of AJs require accurate regulation of DE-cad [38]. We analyzed AJs using the *DE-cad*:*GFP* knock-in allele, which is fully homozygous viable and expresses at the same levels compared to the endogenous DE-cad gene, shotgun (*shg*) [65]. DE-cad:GFP outlines the AJs in *Osi*^*9+15+19*^ mutants and in wild-type control at stage 16 (compare Fig 6B with 6A). At early-stage 17, unlike uniform DE-cad:GFP localization along AJs in wild-type control (Fig 6C), DE-cad:GFP started to show some breaks along the AJs in *Osi*^*9+15+19*^ mutant trachea (arrowheads in Fig 6D). More breaks in each AJ were observed in *Osi*^*9+15+19*^ mutant trachea at mid-stage 17 (arrowheads in Fig 6F) whereas DE-cad:GFP outlines the AJs uniformly in wild type trachea (Fig 6E). Consistent with AJ defects, we observed one or two large gaps in the trachea at late-stage 17 in *Osi*^*9+15+19*^ mutant trachea while the wild type trachea was intact (compare Fig 6G with 6H). As expected, DE-cad:GFP outlines AJs with only a few breaks (Fig 6I) and no gaps were found in *Osi*^*9+15+19*^ mutant trachea with *Osi9* overexpression (Fig 6J).

SJs control the paracellular barrier function of the trachea. To test the barrier function of SJs, we injected 10 KD Dextran-FITC into the body cavity of the late-stage 17 wild type and *Osi*^*9+15+19*^ mutants (n = 20 each). We observed that in > 90% *Osi*^*9+15+19*^ mutant tracheae, the dye did not diffuse into the lumen (arrows in Fig 6L) of the DTs, similar to what we observed in wild-type embryos (arrows in Fig 6K). Similar to wild-type control (asterisks in Fig 6K), the dye was observed in the surrounding tissues in *Osi*^*9+15+19*^ mutants (asterisks in Fig 6L). The intact SJ was further supported by staining with the apical membrane marker Uninflatable (Uif) and the SJ markers Discs Large (DLG), Coracle (Cor) [66], and Kune-kune (Kune) [43]. These marker proteins showed a similar localization pattern in *Osi*^*9+15+19*^ mutant embryos (S3B–S3B’, S3D–S3D’, and S3F–S3F’ Fig) as wild-type control (S3A–S3A’, S3C–S3C’, and S3E–S3E’ Fig). Thus, *Osi* genes are required to maintain AJs but not SJs during tracheal maturation.

### Endosome numbers are reduced in *Osi* mutants

**Fig 7:**
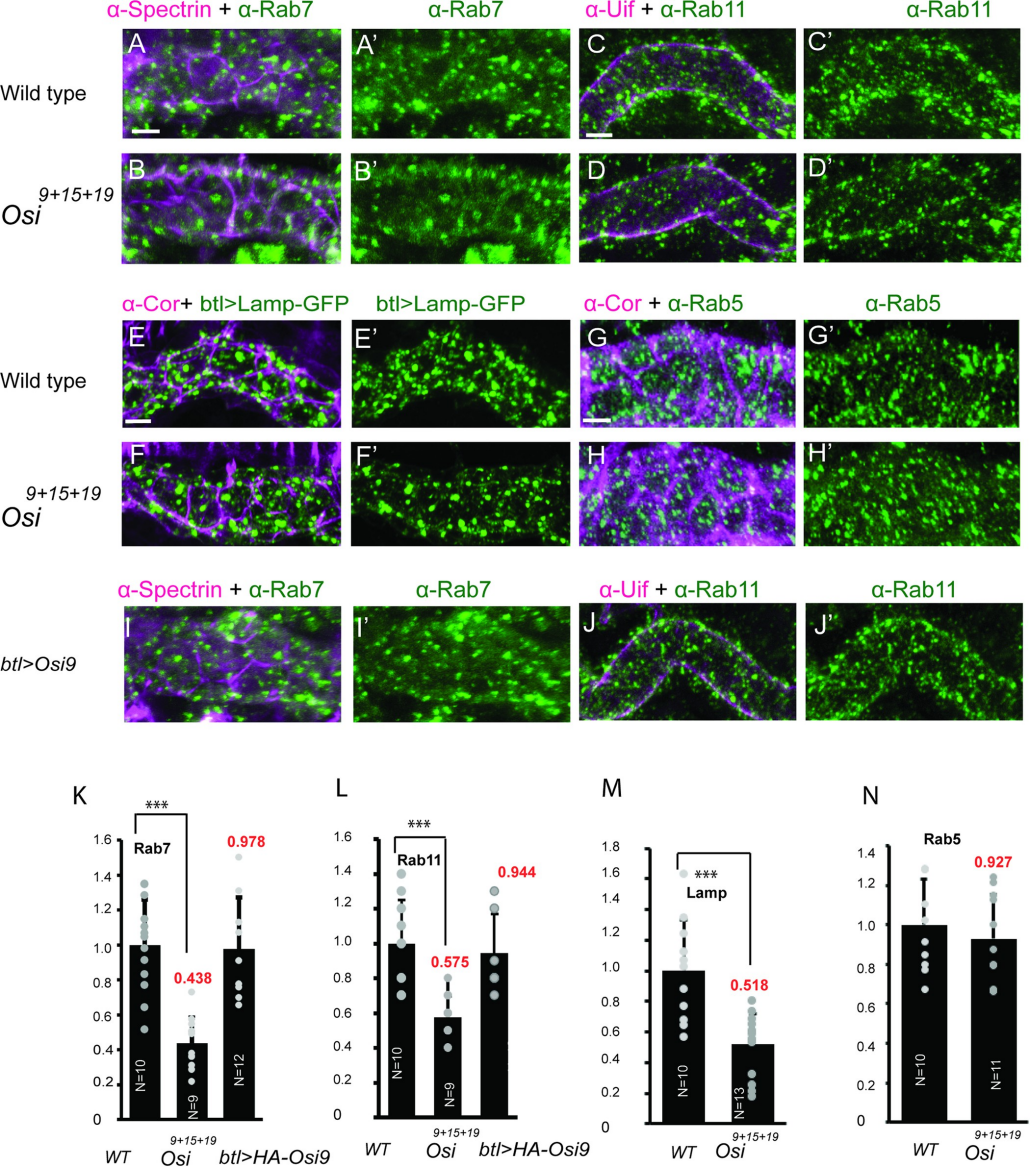
The numbers of late endosomes, lysosomes, and recycling endosomes are significantly reduced in *Osi* mutants. Numbers of endosomes (Rab5, Rab7, Rab11, btl>Lamp-GFP) were analyzed in late-stage 16 wild-type control and *Osi*^*9+15+19*^ mutants. Tracheal cells were outlined by basolateral membrane markers α-Spectrin (purple) and α-Cor (purple) or apical membrane marker α-Uif (purple). Projection of DT tracheal segments 7 (14 sections, 0.5 um step size) was used as a region of interest (ROI). The number of Rab7 punctae (green) was reduced in *Osi*^*9+15+19*^ mutant trachea (B-B’) compared to that in wild-type *w*^*1118*^ trachea (A-A’). The number of recycling endosome Rab11 punctae (green) was reduced in *Osi*^*9+15+19*^ mutants (D-D’) compared to that in wild-type control (C-C’). The number of lysosome btl>Lamp-GFP punctae (green) was reduced in *Osi*^*9+15+19*^ mutant trachea (F-F’) compared to that in wild-type *w*^*1118*^ trachea (E-E’). The number of early endosome Rab5 punctae in wild-type *w*^*1118*^ trachea (G-G’) was comparable to that in *Osi*^*9+15+19*^ mutant trachea (H-H’). Punctae were counted in ROI in N≥9 embryos and normalized to ROI area. The number of Rab7 punctae in *btl-gal4;UAS-HA-Osi9* trachea (I-I) was comparable to that in wild-type control (A-A’). The number of Rab11 punctae in *btl-gal4; UAS-HA-Osi9* trachea (J-J’) was comparable to that in wild-type control (C-C’). (K, L) Quantification of late endosome (Rab7) and recycling endosome (Rab11) numbers in wild-type control, *Osi*^*9+15+19*^ mutant, and *btl-gal4; UAS-HA-Osi9* trachea, respectively. (M, N) Quantification of late endosome (Lamp) and early endosome (Rab5) numbers in wild-type control and *Osi*^*9+15+19*^ mutant trachea, respectively. (L) Error bars, S.E.M. *p < 0.05; **p < 0.005; ***p < 0.0005 Student’s t-test. White lines in A, C, E, G, represented 10um and served as a reference for A-B’ and I-I’, C-D’ and J-J’, E-F’, G-H’ respectively.

Since Osi proteins are colocalized with Rab7-containing late endosomes, Lamp-GFP-containing lysosomes, and Rab11-containing recycling endosomes, we compared the numbers of these endosomes in *Osi* mutants (triple mutant *Osi*^*9+15+19*^ was shown) to wild type control by analyzing endosomes markers in late-stage 16 embryos. We quantified the relative numbers of these endosome punctae in tracheal segment 7 of the wild type and *Osi*^*9+15+19*^ mutants, and the relative numbers were provided (S1 Data). The relative numbers of Rab7 punctae (compare 7A and 7A’ with 7B and 7B’), Rab11 punctae (compare Fig 7C and 7C’ with 7D and 7D’), and Lamp-GFP punctae (compare Fig 7E and 7E’ with 7F and 7F’) in *Osi*^*9+15+19*^ mutants were reduced when compared to wild-type trachea. Nevertheless, no obvious changes to Rab5 were observed (compare Fig 7G and 7G’ with 7H and 7H’). The numbers of Rab7, Rab11, Lamp-GFP, were reduced to 43.8% (Fig 7K), 57.5% (Fig 5L), 51.8.% (Fig 5M) of the wild-type control, respectively, whereas no significant changes were observed in Rab5 (Fig 7N). Thus, the early endosomes were relatively unaltered whereas the maintenance of late endosomes and lysosomes was affected in *Osi* mutants, suggesting possible defects in the degradation of endocytosed molecules. In addition, the reduced Rab11 recycling endosomes suggest that Rab11-mediated apical secretion/recycling was affected in *Osi* mutants. Furthermore, to analyze whether overexpression of *Osi* genes leads to more endosomes, we overexpressed *Osi9* in wild type trachea and compared endosome numbers in *Osi9*-overexpressing trachea with wild-type trachea. The relative numbers were provided (S1 Data). No significant changes of Rab7-containing late endosomes (compare Fig 7I and 7I’ with 7A and 7A’) and Rab11-containing recycling endosomes (compare Fig 7J and 7J’ with Fig 7C and 7C’) were observed (Fig 7K and 7L). Taken together, endosomes (late endosomes, lysosomes, recycling endosomes) were reduced in *Osi* mutants. However, overexpression of *Osi* genes in wild type trachea was not sufficient to increase endosome numbers.

### *Osi* genes regulate tracheal tube maturation potentially through Rab11-mediated trafficking

**Fig 8:**
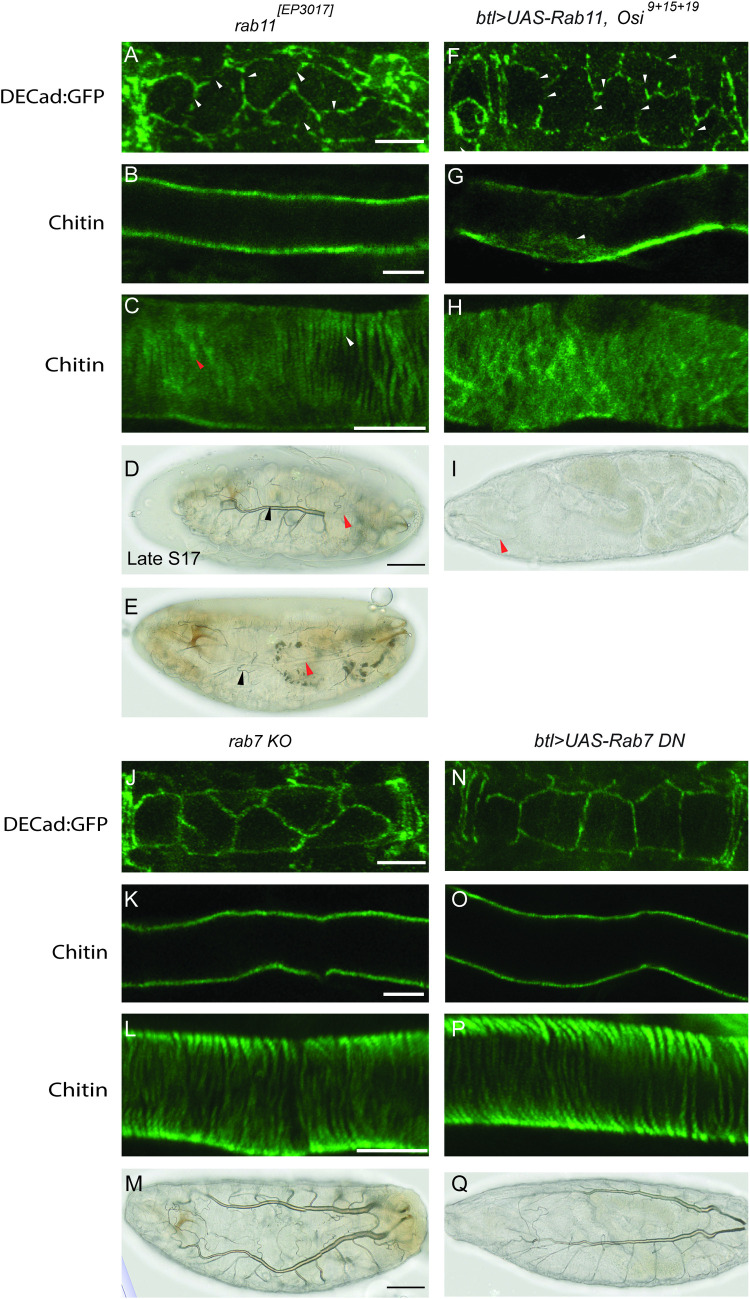
*Osi* genes regulate tube maturation potentially through Rab11- but not Rab7-mediated trafficking. Mid-stage 17 *rab11* mutants (A) showed discontinuous DE-cad:GFP (white arrowheads), (B) a cleared tracheal lumen, (C) inconsistent taenidial fold formation (orange arrowheads indicated disorganized taenidial folds and white arrowheads indicated organized taenidial folds), (D, E) and partial air filling (orange arrowheads indicated tracheal branches lacking air filling and black arrowheads indicated tracheal branches with air filling) at late-stage 17. Overexpression of *rab11* in the triple mutant background (*btl>rab11*, *Osi*^*9+15+19*^) failed to rescue tube maturation defects observed in *Osi*^*9+15+19*^ mutants. (F) discontinuous DE-cad:GFP (white arrowheads), (G) luminal retention of chitin (white arrowhead), (H) disorganized taenidial folds, and (I) lack of air filling (orange arrowhead) were observed. *rab7 KO* mutant and *btl>rab7DN* trachea exhibited (J, N) DE-cad:GFP that outlined AJs, (K, O) a cleared tracheal lumen, and (L, P) taenidial fold formation at mid-stage 17, and (M, Q) air filled trachea at late-stage 17. Taenidial folds were represented in green by the chitin-binding probe (C, H, L, P). Tracheal air filling was depicted in late-stage 17 embryos via standard brightfield microscopy. The white scale bars in A-C and J-L represented 10μm. The black scale bars in D and M represented 50 μm. To analyze DEcad:GFP, mid-stage 17 embryos (18–20 hrs after egg laying) were used. To analyze taenidial folds and lumen clearance, mid-stage 17 embryos (19.5–20 hrs after egg laying) were used. To analyze air filing, late-stage 17 embryos (21.5–22 hrs after egg laying) were used.

The discontinuous DE-cad:GFP along AJs in *Osi* mutants indicates the defective maintenance of AJs. Interestingly, DE-cad is a Rab11-compartment cargo, and maintaining the DE-cad level at the AJs requires Rab11-mediated apical targeting in the *Drosophila* trachea [38,39]. To test whether the Rab11-mediated trafficking contributes to the AJ defect, we analyzed DE-cad:GFP in the zygotic *rab11* amorphic allele, *rab11*^*[EP3017]*^ [67]. We observed discontinuous DE-cad:GFP (Fig 8A) in ~80% of *rab11* mutant trachea (10 embryos analyzed). This phenotype was consistent with what was observed in *Osi* mutants. We also analyzed luminal clearance and taenidial fold formation in *rab11* mutants by immunostaining with the chitin-binding probe. Chitin was cleared from the lumen (Fig 8B) in all *rab11* mutant trachea (12 embryos analyzed). However, taenidial folds were formed in some parts (Fig 8C white arrowheads) of the trachea but disorganized in other parts (Fig 8C orange arrowheads) in ~80% of *rab11* mutant trachea (12 embryos analyzed). In addition, we observed partial air filling in ~80% (46/59 embryos) of these mutants (Fig 8D–8E, black arrowheads point to tubes with air, and orange arrowheads point to tubes without air). The similar tube maturation defects in *Osi* mutant and *rab11* mutant trachea as well as the reduced Rab11-containing recycling endosomes in *Osi* mutant trachea suggest the possibility that *Osi* genes potentially regulate tube maturation through Rab11-mediated trafficking. To test whether this regulation is through the maintenance of endosomes, we overexpressed *rab11* in *Osi*^*9+15+19*^ mutant background and analyzed tube maturation. Unexpectedly, no obvious rescue was observed. Similar to *Osi*^*9+15+19*^ mutant trachea, discontinuous DE-cad:GFP (arrowheads in Fig 8F), minor retention of luminal chitin (arrowhead in Fig 8G), disorganized taenidial folds (Fig 8H), and lack of air filling (orange arrowhead in Fig 8I) were observed. Thus, increasing the number of Rab11-containing endosomes is not sufficient to rescue tube maturation defects in *Osi* mutants. Taken together, these results suggest that *Osi* genes are potentially involved in other aspects of Rab11-mediated trafficking.

The localization of Osi proteins at late endosomes and lysosomes, and the reduction of these endosome numbers in *Osi* mutants prompted us to test whether Rab7 plays a role in *Osi-*regulated tube maturation. We analyzed zygotic *rab7* loss of function mutants (*rab7KO*), *FRT82B rab7Gal4-knock-in/TM3* [68]. However, we observed the uniform localization of DE-cad:GFP along AJs (Fig 8J), lumen clearance (Fig 8K), taenidial folds formation (Fig 8L), and air filling (Fig 8M) in *rab7KO* mutants. To rule out the possibility that the normal tube maturation in *rab7* mutants is due to the maternal contribution, we overexpressed the dominant negative form of *rab7* (*rab7-DN*) in the trachea to discard the role of *rab7*. Similar to *rab7KO* mutants, no obvious defects in AJ (Fig 8N), lumen clearance (Fig 8O), taenidial fold formation (Fig 8P), and air filling (Fig 8Q) were observed. Thus, Rab7 might not play a significant role in tube maturation. However, we cannot rule out the possibility that they might play a minor role.

## Discussion

In this paper, we analyzed the function of three *Osi* gene family members (*Osi9*, *Osi15*, *Osi19*) in the *Drosophila* trachea. Several lines of evidence suggest that these genes have similar functions. First, three Osi proteins have similar intracellular vesicular localization; Second, single mutants of these genes did not show obvious phenotypes, but double mutants and triple mutants showed severe defects including cellular junctions, aECM clearance, taenidial folds formation, tube morphology, and gas filling during tube maturation. Furthermore, all three *Osi* genes at least partially rescued the *Osi*^*9+15+19*^ triple mutant phenotype, suggesting possible functional redundancy of these genes. Nevertheless, variation in the efficiency of rescue existed among these *Osi* genes. This variation is likely not due to the difference in the levels of the expression of the transgenes since these transgenes are expressed at similar levels. However, these transgenes are not inserted in the same genetic locus [51]. Thus, the variation could be due to the different insertion sites of the transgenes, or due to subtle variations in the functions of these genes. Interestingly, the observation that in a few vesicles, not all three Osi proteins are present, suggests minor variations in the expression pattern of these genes.

Tracheal tube maturation is a multi-step process where each step occurs in a strict developmental sequence. It begins with the apical secretion of luminal components (stages 14–16), which forms a chitin-based aECM to control tube diametric expansion and elongation. Then, this first wave of aECM is cleared from the lumen through endocytosis at mid-stage 17. The second wave of aECM, the taenidial folds, starts to develop at late stage 15 and matures at mid-stage 17. The taenidial folds prevent the tube from collapsing, especially after the clearance of the lumen. Thereafter, the liquid is cleared and gas filling follows. Although a majority of the luminal components are cleared from the lumen, residual luminal components are still observed in *Osi* mutants. Nevertheless, this phenotype is relatively minor. Previous work has shown that the Rab5-mediated endocytosis pathway regulates luminal clearance. However, we did not observe changes in Rab5 in *Osi* mutants, and no lumen clearance defect was observed in the *rab7* mutants. These results suggest that the Rab5-mediated endocytosis pathway is not involved in the lumen retention phenotype in *Osi* mutants. However, we cannot rule out the possibility that *Osi* genes are required for complete lumen clearance through Rab5 independent endocytosis or pinocytosis pathway.

In addition to minor lumen retention phenotype, the taenidial folds are not properly formed, tubes are crushed, and air does not fill the lumen in *Osi* mutants. As the taenidial folds provide stiffness to the tube, especially after the lumen clearance, the disorganized taenidial folds could lead to the crushed tubes in *Osi* mutants. Furthermore, the air-filling defect is likely due to either incomplete luminal clearance or abnormal taenidial folds, as defects in either will lead to air inflation defects [61,69]. Taenidial ridges are formed by the extrusion of chitin into the aECM. Genes that affect taenidial folds formation include those controlling: chitin synthesis such as *kkv* and *mummy* (*mmy*) [29,70]; chitin-binding such as *Gasp* and *obstructor-A* (*obst-A*) [26,71]; chitin-modification such as Serp, *verm* [27]; chitin-deposition such as *exp* and *reb* [72]; the secretion of chitin based aECM such as Arf1-COPI secretory pathway component *gartenzwerg* (*garz*) and the COPII complex component *Sec24CD* [73,74]; and the stabilization of the chitin-based aECMs such as *convoluted* (*conv*), *dumpy* (*dp)*, *Mmp1*, and *uninflated* (*uif*) [69,75–77]. Although *Osi* genes are not required for chitin synthesis and secretion, they could be potentially involved in the trafficking of molecules required for aECM modification, stabilization, or currently unknown mechanisms that are required for taenidial fold formation.

The localization of Osi proteins in endosomes (late endosomes, lysosomes, recycling endosomes) suggests the possibility that *Osi* genes regulate tube maturation through endosome-mediated trafficking. We assume that reduced late endosomes and lysosomes in *Osi* mutant trachea could cause defective degradation of endocytosed proteins, which leads to defects in tube maturation. However, we did not observe obvious defects in zygotic *rab7* loss of function mutant trachea. To discard the maternal contribution of *rab7*, we overexpressed *rab7DN* in the trachea, again, no obvious defects were observed. Thus, Rab7 may not be actively involved in tube maturation. However, we cannot rule out the possibility that *rab7DN* does not sufficiently discard the *rab7* function, which prevents us from observing defects. Interestingly, the apical targeting of biosynthetic DE-cad depends on Rab11-containing recycling endosomes to maintain the AJs during tube morphogenesis in the *Drosophila* trachea [38,39]. In addition, DE-cad is mainly recycled through Rab11-positive endosomes in *Drosophila* epithelial tissues, such as the salivary gland and epidermis [78–80]. Moreover, a recent study in *Drosophila* follicular epithelium revealed there are three mechanisms mediating DE-cad accumulation at AJs. One is Rab11 dependent and targets DE-cad directly to the AJs, while the other transports DE-cad to the lateral membrane. Lateral DE-cad either reaches the AJs by RabX1 (mammalian Rab10)-dependent endocytosis and targeted recycling or by an apically directed flow within the plasma membrane [81]. The discontinuous AJ component DE-cad:GFP in *Osi* mutant trachea suggests the possible involvement of Rab11-mediated trafficking in *Osi*-regulated tube maturation. As expected, the discontinuous DE-cad:GFP in zygotic *rab11* mutants was observed, suggesting the involvement of Rab11-mediated trafficking in AJ stability. Furthermore, defects in taenidial fold formation were also observed in *rab11* mutants, indicating the possible involvement of Rab11-mediated trafficking of cargoes in taenidial fold formation.

**Fig 9:**
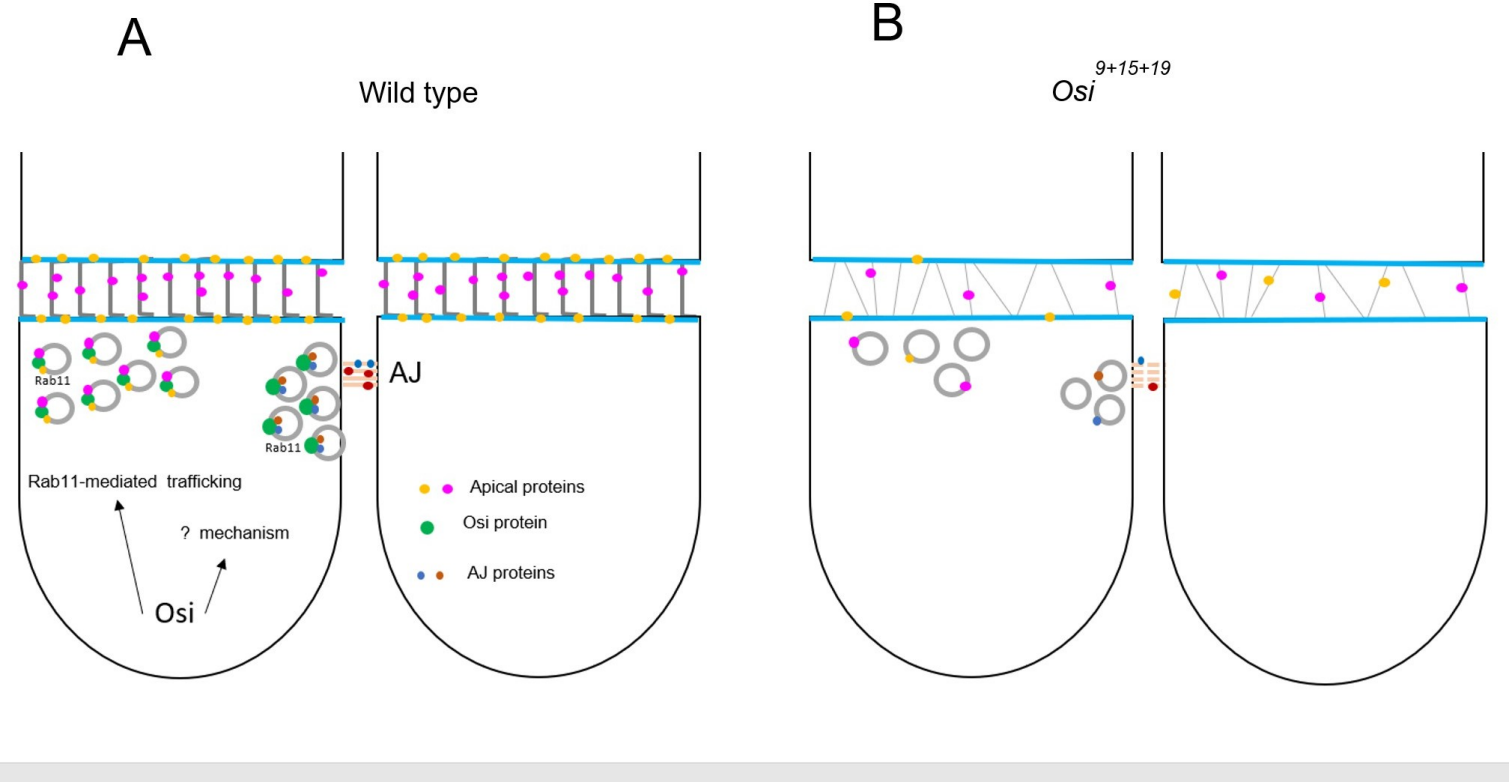
Model of the function of *Osi* genes in the *Drosophila* trachea. *Osi* genes potentially regulate tube maturation through Rab11-mediated trafficking. (A) In wild-type control, Osi proteins are hypothetically involved in sorting cargos to Rab11-containing recycling endosomes and/or in transporting cargos apically to sustain AJs and to form taenidial folds. After apical delivery, Osi proteins could be recycled back to carry out their functions again. (B) In *Osi* mutants, these cargos are not sorted and transported efficiently, which leads to reduced apical delivery of cargos required for tube maturation. It is also possible that *Osi* genes regulate a currently unknown mechanism that converges or acts in parallel with Rab11-mediated trafficking in wild type trachea (A). Green-filled circles are Osi proteins, grey circles are Rab11-containing recycling endosomes. Filled pink and orange circles are cargo proteins delivered to the apical membrane/aECM. Filled blue and dark brown circles are cargo proteins delivered to AJs. Blue lines are apical membranes, and gray lines between apical membranes are taenidial folds. Solid and dotted light brown lines are AJs in wild type and *Osi* mutants respectively.

The similar tube maturation defects observed in *rab11* and *Osi* mutants, and the reduced number of Rab11-containing recycling endosomes suggest that *Osi* genes potentially regulate tube maturation through the maintenance of Rab11-containing endosomes (gray circles in Fig 9A). Surprisingly, increasing Rab11-containing endosomes in the *Osi* mutant trachea was not able to rescue the tube maturation defects. Thus, *Osi* genes are likely involved in other aspects of endosome-mediated trafficking. Taken together, we have proposed a model of the function of *Osi* genes in tube maturation. In wild type trachea (Fig 9A), Osi proteins (filled green circles in Fig 9A) are potentially involved in sorting the “correct” cargoes (filled pink, orange, blue, and dark brown circles in Fig 9A) into the Rab11-containing recycling endosomes, and/or in transporting these cargoes to the AJs for their stability (filled dark brown and blue circles in Fig 9A) as well as to the aECM for taenidial folds formation (filled pink and orange circles in Fig 9A). Osi proteins could be co-transported with these cargoes and then be recycled back to carry out their functions again. In *Osi* mutants (Fig 9B), these cargoes are not effectively sorted and apically transported, which leads to defects in tube maturation, such as taenidial fold formation and AJ stability. However, it is also possible that *Osi* genes regulate a currently unknown mechanism that either converges or acts in parallel with Rab11-mediated trafficking to regulate tube maturation (Fig 9A). To reveal the underlying molecular mechanisms, Osi-interacting proteins will need to be identified.

*Osi* genes are highly conserved in insect species. No obvious homologs in vertebrates have been reported. However, it was mentioned that *Osi* genes show potential gene sequence homology to the *Glyoxalase 1* (*Glo-1*) gene [82]. The Glo-1-dependent glyoxalase system is the major system involved in the detoxification of methylglyoxal, a highly reactive dicarbonyl metabolite of glycolysis [83]. Reduced Glo-1 activity leads to vascular complications [84] and the malfunction of tissues [85]. Little is known about the function of *Glo-1* in vesicular trafficking. The study of *Osi* genes could potentially provide evidence of novel functions for *Osi* functional homologs.

## Materials and methods

### Fly strains

Experiments utilized *w*^*1118*^ as the wild-type control strain. *UAS-Serp-CBD-GFP* line [27] is from Dr. Stephen Lustig. *btl-gal4* line [86] is from Dr. Mark Krasnow. *DE-cad*:*GFP* line [65] is from Dr. Yang Hong. *UAS-rab11* [87] *and UAS-rab7DN* [87] lines are from Dr. Marcos González-Gaitán. *UAS-rab4-YFP*, *UAS-rab7DN-YFP* and *UAS-rab11-GFP* [58], *UAS-Lamp-GFP* [59], *Rab11*^*[EP3017)*^ [67], *FRT82B rab7Gal4-knock-in/TM3* [68], *Drop/TM3GFP* [88], and *P{nos-Cas9*.*R}attP4*0 [89], are from Bloomington stock center. *P{TKO*.*GS01947}attP40* and *P{TKO*.*GS01948}attP40* gRNA lines are from DRSC/TRiP functional genomics resources & DRSC-BTRR *(**https*:*//fgr*.*hms*.*harvard*.*edu/fly-in-vivo-rnai**)*.

### Osi antibodies

Three Osi antibodies were generated in Guinea pig, mouse, and rabbit respectively against specific regions in Osi9, Osi15, and Osi19 proteins, such as peptide HEHSYGRSLPSDDSQ 208-222aa in Osi9, peptide RSFGASKAVESFEDRAE 59-75aa in Osi15, and peptide KDSFQVSNLEVRSNGEKTTP 63-82aa in Osi19. These antibodies were generated at Pocono Rabbit farm.

### CRISPR/Cas9 mutant generation

Single mutants *Osi*^*9*^, *Osi*^*15*^, and *Osi*^*19*^, as well as the double mutant, *Osi*^*15+19*^, and triple mutant, *Osi*^*9+15+19*^, were generated by CRISPR/Cas9 as described in [90]. The single gRNA line (*P{TKO*.*GS01947}attP40)* carrying target sequences of Osi15 (5’ CGGTGTTGTCCTGGGAGGGAAGG 3’) and of Osi19 (5’ CATGGCCCGCTCTTTGATGCAGG 3’) was obtained from DRSC/TRiP functional genomics resources & DRSC-BTRR *(**https*:*//fgr*.*hms*.*harvard*.*edu/fly-in-vivo-rnai**)*. The transgenic flies were then crossed to a *nos-cas9* line on the 2^nd^ chromosome. The resultant progenies were individually crossed to *Drop/TM3*, *twi-GFP* to establish stocks. Roughly 50 independent candidate mutant lines were established. Homozygous mutant individuals were identified by the absence of *GFP* expression from *TM3 P[w*^*+*^*; twi–GFP]*. Sanger sequencing was performed on PCR fragments amplified from genomic DNA around target sites in homozygous mutant embryos to determine the deletion breakpoints. The single mutants, *Osi*^*15*^ and *Osi*^*19*^, as well as the double mutant, *Osi*^*15+19*^, were isolated. PCR was performed to amplify regions between the *Osi15* and *Osi19* genes to confirm and ensure that these regions were not mutated during gene editing. Therefore, these generated mutants only carry deletions in the respective genes and leave other genes located in between them unchanged. A similar approach was used to isolate the *Osi*^*9*^ single mutant using a single gRNA line (*P{TKO*.*GS01948}attP40)* carrying the target sequence of *Osi9* (5’ GTCCATGGTCTTGTGCATGAAGG 3’) and of *CG15887* (CGCCCTCGTCTGCCTCTTTTTGG) to cross the nos-cas9 line. The single mutant, *Osi*^*9*^ with intact *CG15887* was isolated. In addition, PCR was performed to amplify regions between the *Osi9* and *CG15887* genes to ensure that these regions were not mutated during gene editing. The triple mutant, *Osi*^*9+15+19*^, was isolated using gRNA carrying the targeting sequences of *Osi15* and *Osi19* to cross nos-cas9 into the *Osi*^*9*^ mutant background. The deletion breakpoints for the triple mutant were similarly determined by Sanger sequencing PCR fragments amplified from genomic DNA in homozygous triple mutant embryos. We also amplified the regions between Osi15 and Osi19 to ensure that these regions were intact.

### Embryo collection

Embryos were collected and aged at 25°C degree. Stage 16 embryos (14–16 hrs after egg laying), early-stage 17 embryos (16–18 hrs after egg laying), mid-stage 17 embryos (18–20 hrs after egg laying), and late-stage 17 embryos (20–22 hrs after egg laying) were prepared by collecting embryos for 2 hrs and aged for 14 hrs, 16 hrs, 18 hrs, and 20 hrs respectively. Late-stage 16 embryos (15.5–16 hrs after egg laying) were prepared by collecting embryos for 30min and aged for 15.5 hrs.

To analyze luminal clearance and taenidial folds formation, mid-stage 17 embryos (19.5–20 hrs after egg laying) were prepared by collecting embryos for 30min and aged for 19.5 hrs to ensure the lumen was cleared and taenidial folds were formed since luminal clearance occurs around 18hrs 40min (±20min) after egg laying [19]. To analyze air filling, late-stage 17 embryos (21.5–22 hrs after egg laying) were prepared by collecting embryos for 30min and aged for 21.5 hrs to ensure that air filling occurs.

### Immunohistochemistry

Embryo staining: Embryos of control and mutants were fixed simultaneously with 4% formaldehyde for 20min. These embryos were subjected to immunostaining after fixation. For immunostaining, embryos were washed in PT (1xPBS, 0.1% Triton X-100) three times each for 10 minutes and blocked in PBT-NGS (1xPBS, 0.1% BSA, 0.1% Triton X-100, 5% Normal Goat Serum (NGS)) for 30 minutes. For immunostaining the mid-stage 17 embryos, embryos were permeabilized by incubating for 1h at RT in a blocking solution with Saponin [(0.5%w/v BSA in PBST/S (0.2%w/v Saponin)]. Embryos were incubated in primary antibodies which were diluted in PBT-NGS overnight at 4°C, washed 3 times each for 10 minutes, then 3 times each for 20 minutes in PBT, and blocked for an additional 30 minutes in PBT-NGS. Embryos were then incubated in secondary antibodies that were diluted in PBT-NGS (1:200) overnight at 4°C. Following incubation in secondary antibodies, samples were washed 3 times each for 10 minutes, then 3 times each for 20 minutes in PBT. The remaining PBT was removed, and embryos were mounted in SlowFade Diamond Antifade Mountant (Fisher scientific). Embryos of control and mutants were prepared the same way and images were taken within the same day with the same confocal setting.

### Antibodies used for immunostaining

The chitin-binding probe was prepared in our laboratory as described [91]. Chitin-546 was used at 1:10 dilution. The following primary antibodies were used: Chicken anti-GFP (1:200) (Abcam), Guinea Pig anti-Uif (1:300) [69], Mouse anti-Gasp (1:5) (DSHB), Guinea Pig anti-Cor (1:500) [66], Rabbit anti-Verm (1:100) [27], Rabbit anti-Kune-kune (1:200) [43], Mouse anti-DLG (1:1) (DSHB), Mouse anti-Spectrin (1:10) (DSHB), Mouse anti-Cnx99A (1:5) (DSHB), Rabbit anti-GM130 (1:200) (Abcam), Mouse anti-KDEL (1:200) (Abcam), Rabbit anti-Rab5 (1:200) (Abcam), Rat anti-Rab11 (1:100) [92], and Mouse anti-Rab7 (1:10) (DSHB). Antibodies generated in this study: Rabbit anti-Osi19 (1:300), Rat anti-Osi15 (1:25), Guinea Pig anti-Osi9 (1:10).

The following secondary antibodies (Invitrogen life technologies) were used: AlexaFluor 488 anti-chicken IgG, anti-rat IgG, anti-mouse IgG, anti-rabbit IgG, anti-guinea pig IgG; AlexaFluor 543 anti-rat IgG, anti-rabbit IgG, anti-guinea pig IgG, anti-mouse IgG; AlexaFluor 594/546 anti-mouse IgM; AlexaFluor 647 anti-rat IgG, anti-mouse IgG, anti-rabbit IgG, anti-guinea pig IgG; AlexaFluor 647 plus anti-mouse, anti-rabbit; AlexaFluor 488 plus anti-mouse, anti-rabbit), and AlexaFluor 405 anti-Guinea Pig. All secondary antibodies were used at 1:200 dilution.

### Image acquisition

Images were obtained using a Nikon Eclipse Ti C2+ confocal microscope with the same confocal setting for mutant and wild-type embryos. Confocal Z-stacks were obtained with the 63x NA 1.24 oil objective with image format ≥2048 × 2048, pinhole size 0.7 AU. Z-step sizes of 0.5 μm were used.

### Co-localization of Osi protein and endosomes

The quantification of the colocalization of endosome and Osi proteins was performed using NIS-Elements AR. Tracheal cells of the seventh dorsal trunk metamere were used as regions of interest (ROI). A total of 3 embryos were used for each comparison. Five z-sections (0.2 μm step size) of the seventh dorsal trunk metamere in a single embryo were denoised via the NIS-Elements denoising feature before measuring Pearson’s correlation coefficient and colocalization analysis. Binary areas and thresholds within the regions of interest were determined that exclude nonspecific staining signals. Colocalization was determined through the overlapping binary area of two immunostaining signals, or "Intersection". The percent of colocalization was determined by dividing the binary areas of the Intersection binary layer by the Osi protein’s binary area.

### Vesicle quantification

Max Intensity projections of 14 sections (0.5 μm step size) of tracheal segment 7 were obtained as a region of interest (ROI). Fiji software was used to analyze the numbers of endosome punctae. The subtraction background tool and the threshold tool were used to create binarized masks of the vesicles. The number of vesicles was counted using the Analyze Particles tool and the parameters were set to 0.5–2.5μm^2^ size, 0–1 circularity; a mask of the result was obtained. Punctae numbers were then normalized with the total area of the ROI. Each embryo’s ratio of endosome numbers per unit area was then divided by the wild-type average, and a Student’s two-tailed t-test was used to calculate the p-value.

The work has complied with all relevant ethical regulations for animal research. Because *D*. *melanogaster* is an invertebrate species, no institutional approval of experimental protocols was required.

## Supporting information

S1 Fig The expression of Osi proteins in *Osi* loss of function mutants. The expression of Osi9, Osi15, and Osi19 were analyzed in stage 16 *Osi*^*9*^, *Osi*^*15*^, *Osi*^*19*^, *Osi*^*15+19*^ double mutant, and *Osi*^*9+15+19*^ triple mutant embryos by immunostaining. Meanwhile, the tracheal lumen was labeled using a chitin probe. DT tracheal segments were shown. (A-A”’) The Osi9 (green in A’), Osi15 protein (blue in A”), and Osi19 protein (purple in A”’) were expressed in vesicles in all tracheal cells of wild-type embryos. (B-B”’) Osi9 (green in B’) was not expressed in *Osi*^*9*^ mutant embryos. (C-C’”) Osi15 was not expressed (blue in C”) in *Osi*^*15*^ mutant embryos. (D-D’”) Osi19 (purple in D’”) was not expressed in *Osi*^*19*^ mutant embryos. (E-E’”) Osi15 (blue in E”) and Osi19 (purple in E’”) were not expressed in *Osi*^*15+19*^ double mutants. (F-F’”) Osi9 (green in F’), Osi15 (blue in F”), and Osi19 (purple in F’”) were not expressed in *Osi*^*9+15+19*^ triple mutants. The tracheal luminal matrix was represented by white in A-F merged images (the white bar in B represented 10um.)
(TIF)

S2 Fig Individual HA-tagged *Osi* genes are expressed at similar levels in *Osi* mutant trachea. Individual HA-tagged *Osi* genes (*HA-Osi9*, *HA-Osi15*, *HA-Osi19*) were expressed in *Osi* mutant trachea by *btl-gal4* respectively. The expression of HA-Osi9 (red in A), HA-Osi15 (red in B), and HA-Osi19 (red in C) proteins were analyzed in stage 16 embryos using immunostaining with anti-HA antibodies. Dorsal trunk segments were shown. The white scale bar in A represented 10μm.
(TIF)

S3 Fig Tracheal SJs are intact in *Osi* mutants. SJ components of wild-type *w*^*1118*^ and *Osi* ^*9+15+19*^ triple mutant tracheal tubes in stage 16 embryos were analyzed with antibodies against the SJ proteins DLG, Kune, and Cor. Tracheal apical membrane marker Uif was shown in purple (A-F). SJ components DLG (green in B-B’), Kune (green in D-D’), Cor (green in F-F’) in *Osi*^*9+15+19*^ triple mutant trachea showed similar localization to DLG (green in A-A’), Kune (green in C-C’), Cor (green in E-E’) in wild type trachea respectively. White scale bar in A represented 10μm and served as a reference for A-F’.
(TIF)

S1 Data Quantification of endosomes in Fig 7. Relative **n**umbers of endosomes (Rab5, Rab7, Rab11, btl>Lamp-GFP) were analyzed in late-stage 16 wild-type control, *Osi*^*9+15+19*^ mutants, and *btl>Osi9* embryos. The relative numbers of endosomes were normalized against the average numbers in control, which was set as 1. The relative numbers of Lamp in control and *Osi* mutants were shown in the pink area. The relative numbers of Rab7 in *Osi* mutants, wild type control, and *btl>Osi9* embryos were shown in yellow area. The relative numbers of Rab11 in *Osi* mutants, wild type control, and *btl>Osi9* embryos were shown in green area. The relative number of Rab5 in control and *Osi* mutants were shown in blue area.
(XLSX)
