# Supplementary figures and images for: The Osiris family genes function as novel regulators of the tube maturation process in the Drosophila trachea
Source: PLoS Genet. 2023 Jan 23;19(1):e1010571. doi: 10.1371/journal.pgen.1010571 (PMC9870157; doi:10.1371/journal.pgen.1010571)

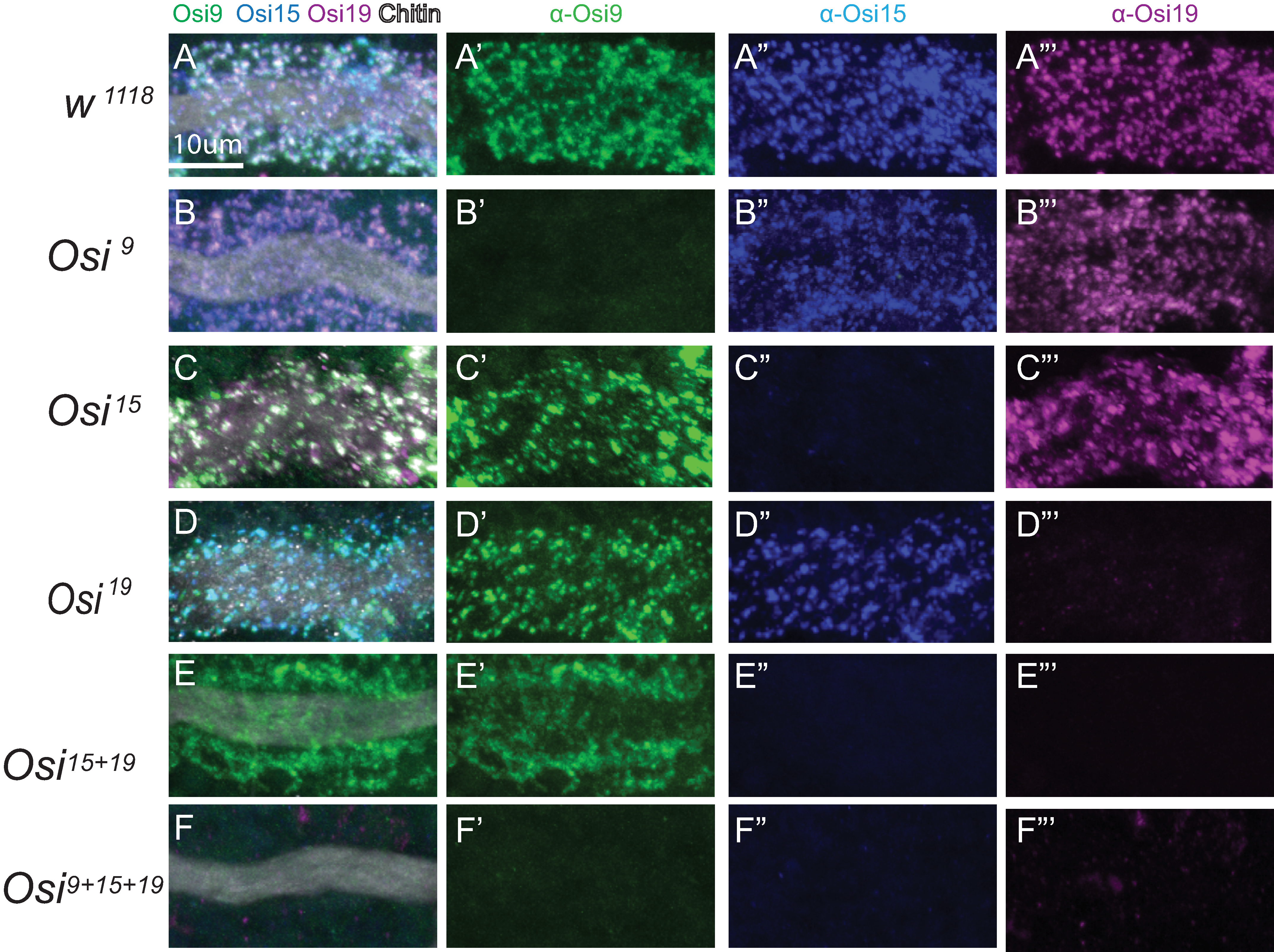

Supplement: S1 Fig — The expression of Osi9, Osi15, and Osi19 were analyzed in stage 16 Osi9, Osi15, Osi19, Osi15+19 double mutant, and Osi9+15+19 triple mutant embryos by immunostaining. Meanwhile, the tracheal lumen was labeled using a chitin probe. DT tracheal segments were shown. (A-A”’) The Osi9 (green in A’), Osi15 protein (blue in A”), and Osi19 protein (purple in A”’) were expressed in vesicles in all tracheal cells of wild-type embryos. (B-B”’) Osi9 (green in B’) was not expressed in Osi9 mutant embryos. (C-C’”) Osi15 was not expressed (blue in C”) in Osi15 mutant embryos. (D-D’”) Osi19 (purple in D’”) was not expressed in Osi19 mutant embryos. (E-E’”) Osi15 (blue in E”) and Osi19 (purple in E’”) were not expressed in Osi15+19 double mutants. (F-F’”) Osi9 (green in F’), Osi15 (blue in F”), and Osi19 (purple in F’”) were not expressed in Osi9+15+19 triple mutants. The tracheal luminal matrix was represented by white in A-F merged images (the white bar in B represented 10um.) (TIF) [file pgen.1010571.s001.tif]

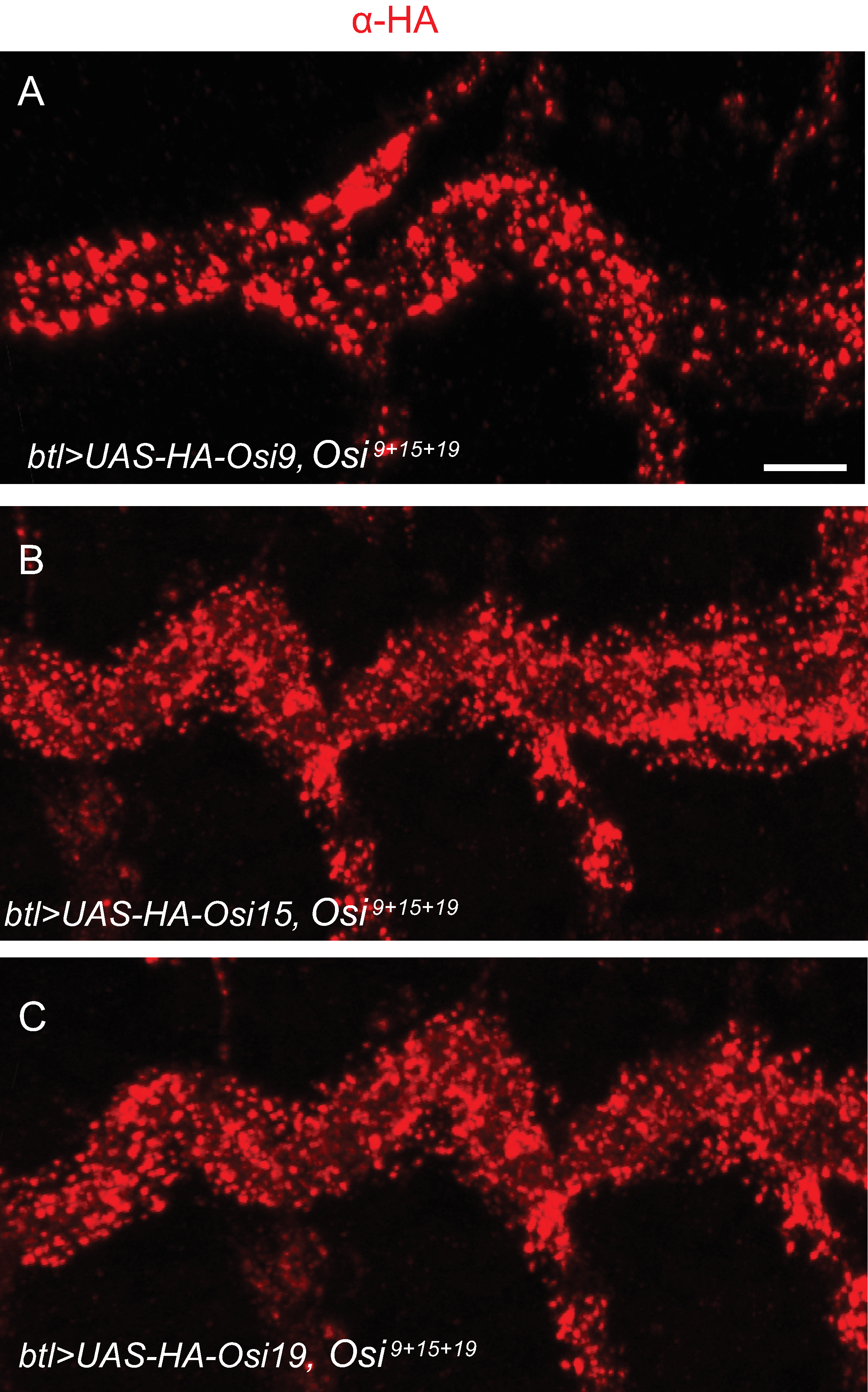

Supplement: S2 Fig — Individual HA-tagged Osi genes (HA-Osi9, HA-Osi15, HA-Osi19) were expressed in Osi mutant trachea by btl-gal4 respectively. The expression of HA-Osi9 (red in A), HA-Osi15 (red in B), and HA-Osi19 (red in C) proteins were analyzed in stage 16 embryos using immunostaining with anti-HA antibodies. Dorsal trunk segments were shown. The white scale bar in A represented 10μm. (TIF) [file pgen.1010571.s002.tif]

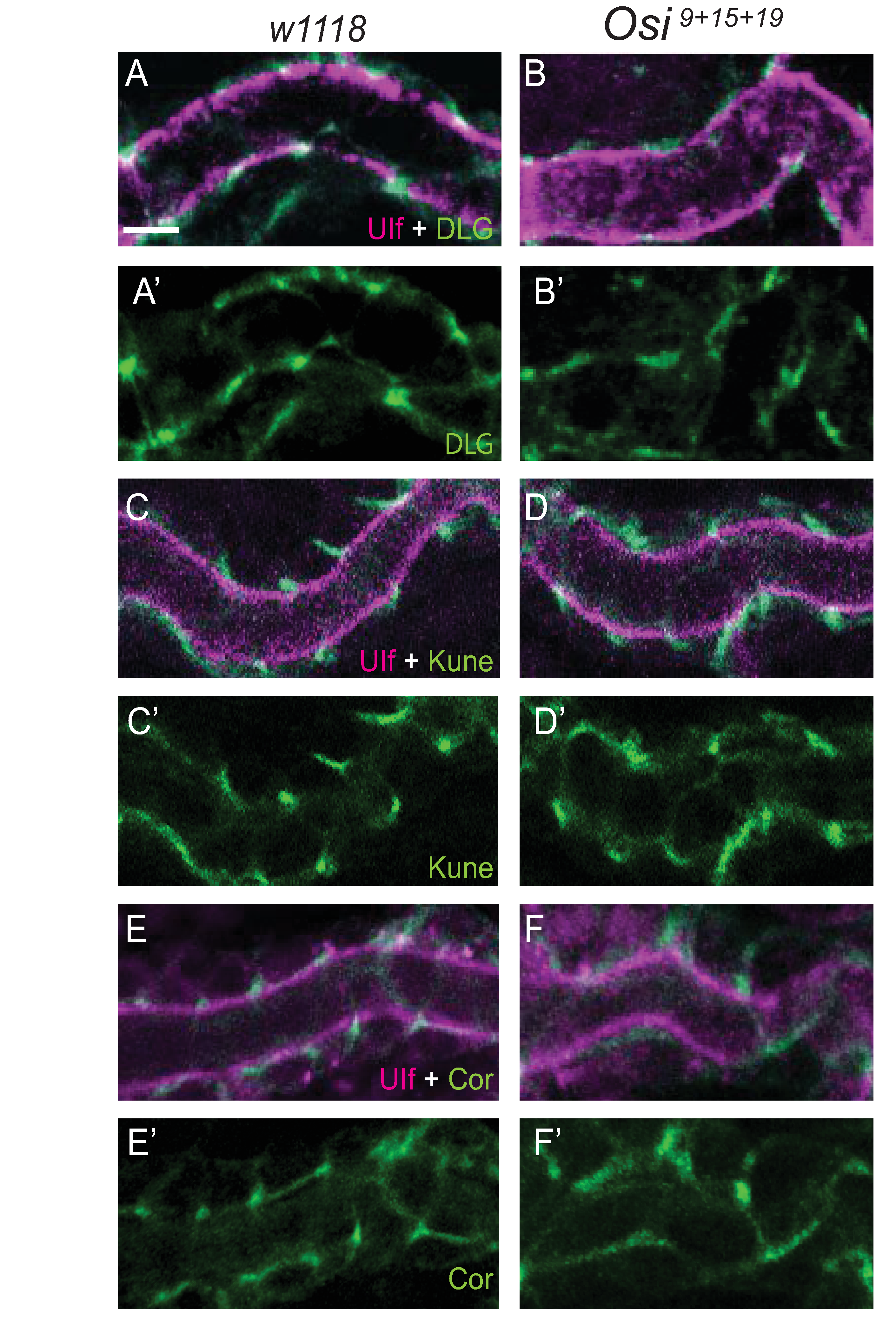

Supplement: S3 Fig — SJ components of wild-type w1118 and Osi 9+15+19 triple mutant tracheal tubes in stage 16 embryos were analyzed with antibodies against the SJ proteins DLG, Kune, and Cor. Tracheal apical membrane marker Uif was shown in purple (A-F). SJ components DLG (green in B-B’), Kune (green in D-D’), Cor (green in F-F’) in Osi9+15+19 triple mutant trachea showed similar localization to DLG (green in A-A’), Kune (green in C-C’), Cor (green in E-E’) in wild type trachea respectively. White scale bar in A represented 10μm and served as a reference for A-F’. (TIF) [file pgen.1010571.s003.tif]
